# Tolerance induction through non-avoidance to prevent persistent food allergy (TINA) in children and adults with peanut or tree nut allergy: rationale, study design and methods of a randomized controlled trial and observational cohort study

**DOI:** 10.1186/s13063-022-06149-4

**Published:** 2022-03-28

**Authors:** Valérie Trendelenburg, Sabine Dölle-Bierke, Nathalie Unterleider, Aikaterina Alexiou, Birgit Kalb, Lara Meixner, Stephanie Heller, Susanne Lau, Young- Ae Lee, Florent Fauchère, Julian Braun, Magda Babina, Sabine Altrichter, Till Birkner, Stephanie Roll, Josefine Dobbertin-Welsch, Margitta Worm, Kirsten Beyer

**Affiliations:** 1 Department of Pediatric Respiratory Medicine, Immunology and Critical Care Medicine, Charité – Universitätsmedizin Berlin, corporate member of Freie Universität Berlin and Humboldt-Universität zu Berlin, Augustenburgplatz 1, 13353 Berlin, Germany; 2 Division of Allergy and Immunology, Department of Dermatology, Venerology and Allergy, Charité – Universitätsmedizin Berlin, corporate member of Freie Universität Berlin and Humboldt-Universität zu, Berlin, Germany; 3 Max Delbrück Center For Molecular Medicine in the Helmholtz Association, Charité - Universitätsmedizin Berlin, corporate member of Freie Universität Berlin and Humboldt-Universität zu Berlin, Berlin, Germany; 4 Si-M / “Der Simulierte Mensch” a science framework of Technische Universität Berlin and Charité - Universitätsmedizin Berlin, Berlin, Germany; 5 Berlin Institute of Health at Charité - Universitätsmedizin Berlin, BIH Center for Regenerative Therapies, Berlin, (BCRT) Germany; 6 Division of Dermatological Allergy, Department of Dermatology, Venerology and Allergy, Charité – Universitätsmedizin Berlin, corporate member of Freie Universität Berlin and Humboldt-Universität zu Berlin, Berlin, Germany; 7 Universitätsklinikum für Dermatologie und Venerologie, Kepler Uniklinikum, Linz, Austria; 8 Experimental and Clinical Research Center, a cooperation between the Max-Delbrück-Center for Molecular Medicine in the Helmholtz Association and the Charité - Universitätsmedizin, Berlin, Germany; 9 Charité – Universitätsmedizin Berlin, corporate member of Freie Universität Berlin and Humboldt-Universität zu Berlin, Experimental and Clinical Research Center, Lindenberger Weg 80, 13125 Berlin, Germany; 10 Institute for Epidemiology, Social Medicine and Health Economics, Charité – Universitätsmedizin Berlin, corporate member of Freie Universität Berlin and Humboldt-Universität zu Berlin, Berlin, Germany

**Keywords:** Protocol, Randomized controlled trial, Peanut allergy, Tree nut allergy, Elimination diet, Higher threshold, Oral tolerance, Persistent food allergy

## Abstract

**Background:**

Peanuts (PN) and tree nuts (TN) are among the most frequent elicitors of food allergy and can lead to life-threatening reactions. The current advice for allergic patients is to strictly avoid the offending food independently of their individual threshold level, whereas sensitized patients without allergic symptoms should frequently consume the food to avoid (re-)development of food allergy. The aim of this trial is to investigate (I) whether the consumption of low allergen amounts below the individual threshold may support natural tolerance development and (II) to what extent regular allergen consumption in sensitized but tolerant subjects prevents the (re-)development of PN or TN allergy.

**Methods:**

The TINA trial consisting of (part I) a randomized, controlled, open, parallel group, single-center, superiority trial (RCT), and (part II) a prospective observational exploratory cohort study. Children and adults (age 1–67 years) with suspected or known primary PN and/or TN allergy will undergo an oral food challenge (OFC) to determine their clinical reactivity and individual threshold. In the RCT, 120 PN or TN allergic patients who tolerate ≥100 mg of food protein will be randomized (1:1 ratio) to consumption of products with low amounts of PN or TN on a regular basis or strict avoidance for 1 year. The consumption group will start with 1/100 of their individual threshold, increasing the protein amount to 1/50 and 1/10 after 4 and 8 months, respectively. The primary endpoint is the clinical tolerance to PN or TN after 1 year assessed by OFC. In the cohort study, 120 subjects sensitized to PN and/or TN but tolerant are advised to regularly consume the food and observed for 1 year. The primary endpoint is the maintenance of clinical tolerance to PN and/or TN after 1 year assessed by challenging with the former tolerated cumulative dose.

**Discussion:**

This clinical trial will help to determine the impact of allergen consumption versus avoidance on natural tolerance development and whether the current dietary advice for PN or TN allergic patients with higher threshold levels is still valid.

**Trial registration:**

German Clinical Trials Register; ID: DRKS00016764 (RCT), DRKS00020467 (cohort study). Registered on 15 January 2020, http://www.drks.de.

**Supplementary Information:**

The online version contains supplementary material available at 10.1186/s13063-022-06149-4.

## Background

Food allergy affects up to 8% of children and 5% of adults in industrialized countries. Peanuts and tree nuts (hazelnuts, walnuts, cashews, almonds, pecan, pistachio, Brazil nuts, and macadamia nuts) are among the most frequent elicitors of food allergic reactions [1–3]. While most of the patients with cow’s milk and hens’ egg allergy gain oral tolerance against these allergens within the first years of life [4, 5], peanut and tree nut allergy usually persist into adulthood. Only about 20% of peanut and about 10% of tree nut-allergic patients develop oral tolerance later in life [6, 7]*.* Peanut and tree nuts can lead to severe, life-threating reactions in patients and are the major elicitors of food-induced anaphylaxis in children and adults [8, 9].

Independent of their eliciting dose, patients with food allergy are currently advised to maintain a strict elimination diet and to carry self-injectable epinephrine at all times in order to use it in case of an accidental reaction [10–12]. However, a strict elimination diet needs a high level of nutritional education and is difficult to maintain due to the ubiquitous use of various food allergens in the food industry. The permanent vigilance and the fear of experiencing allergic reactions of unpredictable severity have a major impact not only on the food choice but also on quality of life of these patients, as well as their caregivers [13]. Hence, effective strategies to prevent persistent food allergy other than the current management recommendation of strict avoidance would be of major importance.

In recent years, in the fields of primary and secondary prevention as well as in the field of treatment of food allergy, there has been a shift away from the concept of avoidance of food allergens towards food allergen exposure [14–17]. While in current management guidelines for food allergy strict avoidance plays the key role to protect the patient from allergic reactions, oral immunotherapy with a controlled specific exposure with food allergens has been studied as a potential effective treatment strategy especially for peanut allergy [18–21] and is today an approved treatment by the FDA and EMA. However, trials on treatment options for various tree nuts are still lacking. Furthermore, most of trials on peanut oral immunotherapy focus on allergic patients who react to very small amounts of peanut (i.e., low threshold levels, ≤ 100 mg or ≤ 300 mg of peanut protein), but many peanut-allergic patients experience symptoms at higher levels only. Some peanut-allergic patients indeed react to very small amounts of peanut [22]. However, in a large retrospective survey on open peanut challenges from the UK, Ireland, and Australia with 1634 peanut challenged children aged 1–18 years, 532 children had a positive peanut challenge [23]. Of those, more than one third of children (38%) tolerated the equivalent of one peanut.

Next to the amount of allergen that triggers an allergic reaction (threshold dose), also the severity of an allergic reaction differs from patient to patient. Currently, the individual threshold dose is not taken into account in the dietary management of the patient. All patients receive the same advice of strict allergen avoidance (10-11). As underlying immunological mechanisms of tolerance development are still unknown, it could be hypothesized that a strict elimination diet with complete allergen avoidance may even promote the persistence of food allergy. There are no studies that systematically investigate the effect of strict avoidance on the persistence of food allergy. The international survey on peanut challenges described above showed that anaphylaxis, particularly to small amounts of peanut, was more common in older children [23]. This suggests that a long period of strict avoidance of peanut through childhood may be a factor leading to an increased clinical reactivity and severity.

Moreover, after having passed an oral food challenge (OFC) without any allergic reactions (negative OFC), regular consumption of the food seems to prevent the clinically tolerant patient from future allergic reactions. However, in daily clinical practice, up to 30% of patients defined as tolerant after an OFC do not (re-)introduce the food in their diet on a regular basis or even continue avoiding the food for various reasons, e.g., dislike, aversion, fear of reactions, or social/cultural/family habits [24–28]. Peanut and hazelnut are the most common foods that are not (re-)introduced into the diet [25, 28]. Prospective surveys, retrospective analysis, and case reports show that some of the patients who continue avoidance or irregular consumption after a negative food challenge do (re-)develop food allergic reactions [24–28]. Hence, we do not know whether sensitized patients with a negative OFC are indeed tolerant or would react only to higher amounts of the allergen or in combination with trigger factors while regular exposure is mandatory to maintain their oral tolerance.

So far, no studies exist systematically investigating the effect of a liberated diet (ingestion of low allergen amounts, below those causing allergic symptoms) compared to strict avoidance in patients with primary peanut and tree nut allergy. Two UK case series with six and 16 peanut-allergic children, respectively, with reactions to peanut at high levels reported on successful introduction of small, gradually increasing quantities of peanuts several times a week at home using regular food products containing peanuts [29, 30].

The aim of the TINA (Tolerance induction through non-avoidance to prevent persistent food allergy) study is to assess whether the introduction of small amounts of peanuts and tree nuts (below the individual threshold level) in children and adults with challenge-proven primary peanut or tree nut allergy promotes natural tolerance development after 1 year compared to strict avoidance. A further aim is to assess possible (re-)development of allergic reactions in peanut and tree nut sensitized patients, who passed the OFC without clinical reactions.

This is an abridged protocol based on protocol version 5.0 dated 09 September 2021. The full protocol is available on: German clinical trials register http://www.drks.de (DRKS00016764, part I randomized controlled trial; DRKS00020467, part II cohort study). The completed Standard Protocol Items: Recommendations for Interventional Trials (SPIRIT) Checklist is included (see Additional file 1) [31].

## Methods

### Objectives

The primary objective of part I (RCT) of the TINA trial is to investigate the impact (superiority) of a liberated diet below the individual threshold level versus complete allergen avoidance on natural tolerance development in children and adults with peanut or tree nut allergy with a higher threshold level (≥100 mg food protein). Secondary objectives are to investigate the impact of a liberated diet below the individual threshold level versus complete allergen avoidance on the threshold level upon OFC and to assess the immunological effect and safety of both dietary interventions. Furthermore, the impact on quality of life, eating habits, and burden of dietary behavior will be assessed.

The primary objective of part II (exploratory cohort study) of the TINA trial is to investigate the impact of regular allergen consumption on the (re-)development of peanut or tree nut allergy in sensitized children and adults with tolerance to peanut and/or tree nut. Secondary objectives are to assess the immunological effect, dietary compliance, and impact on quality of life of a regular allergen consumption.

### Trial design

The TINA trial consists of the following:

- Part I: a randomized, controlled, open, parallel group, single-center, superiority trial (RCT) in peanut or tree nut-allergic patients
- Part II: a prospective observational exploratory cohort study of sensitized but peanut and/or tree nut-tolerant subjects.

**Fig. 1:**
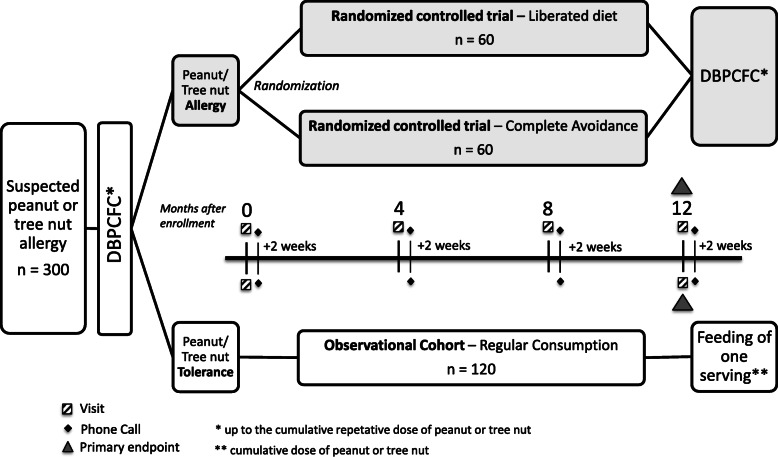
The study diagram shows the flow of the screened subjects (1–67 years of age) after the initial double-blind, placebo-controlled food challenge (DBPCFC) into the randomized controlled trial (allergic participants) or the cohort study (tolerant participants). Subjects of the randomized controlled trial will be randomized 1:1 in the liberated diet group (allergen consumption below the individual threshold level) or the complete allergen avoidance group. After 12 months, all participants will be re-assessed for clinical tolerance development or maintenance of tolerance respectively

All eligible participants will undergo an initial OFC to determine the inclusion into the RCT (allergic participants) or the cohort study (tolerant participants) (Fig. 1). Subjects of the RCT will be randomized 1:1 in the liberated diet group or the complete allergen avoidance group. Subjects of the cohort study are advised to consume the food on a regular basis. After 12 months, participants will be re-assessed for natural tolerance development (part I) or maintenance of tolerance (part II), respectively.

TINA is part of the clinical research unit 339 “Food Allergy and Tolerance” (FOOD@). Using biological samples obtained within the trial, several mechanistic subprojects will be performed in order to gain a deeper understanding of the immunological mechanisms involved in food allergy and tolerance development. In particular, the role of the gastrointestinal as well as skin microbiome, the IgEome, epigenetic mechanisms, allergen-specific B and T lymphocytes, and immunological signatures in the peripheral myeloid compartment, and serological biomarkers will be investigated. Additionally, these different feature spaces will be integrated to gain a more systemic insight and elucidate potential interplay by using systems medicine approach. The methods of these subprojects will be described elsewhere.

### Setting

This single-center trial is conducted at the Charité – Universitätsmedizin Berlin, Germany. Participants are recruited from the Pediatric and Dermatology department of this tertiary care center. Furthermore, recruitment from the general community includes advertisement on hospital websites, via the German Allergy and Asthma Association, social media and distribution of posters and flyers. Screening and all visits throughout the study will take place either in the inpatient or outpatient clinic or clinical research facility.

### Participants

Children and adults (between 1 and 67 years of age) with a suspected or known primary peanut and/or tree nut (hazelnut, cashew, or walnut) allergy planning to undergo an oral food (re-)challenge will be informed about the study. The study population of the RCT and the cohort study each consists of 120 patients with challenge-proven peanut and/or tree nut allergy, aiming to include 40–60% children and at least 40% adults.

#### Inclusion criteria

• Age 1–67 years

• Suspected or known primary peanut and/or tree nut (hazelnut, cashew or walnut) allergy

• Signed informed consent (according to age: participant or parent/legal guardian)

#### Exclusion criteria

• Other severe diseases (cardiac, cystic fibrosis, congenital)

• Participation in another interventional trial that requires consumption of food protein

• Immunotherapy or therapy with biologicals for the treatment of food allergy in the past 12 months

• Uncontrolled asthma

• Usage of antihistamines 3–5 days before OFC

• Therapy with beta-blocker and/or ACE inhibitors

• Systemic immunosuppression

• Pregnancy or lactation

Further exclusion criterion for the RCT is an allergic reaction to less than the 5th dose of peanut or tree nut protein on OFC at screening. The 5th dose is around 300 mg depending on the individual allergen. Dose steps and the corresponding allergen amounts used for OFCs with peanut or individual tree nuts are displayed in Appendix of the full protocol.

Another exclusion criterion for the cohort study is a positive OFC with peanut and/or tree nut at screening.

### Consent procedure

All participants (according to age: participant or parent/legal guardian, referred to participant(s) from here on) must read, sign, and date the informed consent form (see Additional files 2 and 3) approved by the ethics committee before entering the study or undergoing any study-specific procedures. Before consent is given, the investigator or his/her representative will explain verbally the aim, method, source of funding, and the anticipated benefits and potential risks to the participants and answer all questions regarding the study. A unique participant number will be allocated to each participant and assigned chronologically prior to proceeding with study screening. The sequential identification numbers rather than names will be used to collect, store, and report participant information.

### Interventions (RCT)

Peanut and/or tree nut-allergic patients randomized in the “liberated diet group” will get instruction by a dietician/nutritionist on standard peanut and/or tree nut avoidance, but will be allowed to eat small amounts of peanuts or tree nuts at least 3 times per week, preferably daily, starting with 1/100 of their individual threshold dose upon OFC at screening. In addition to verbal education, they will receive written guidelines including a list of commercially available products giving amounts allowed to consume. The patients of the liberated diet group will get their first PN or TN “meal” under medical supervision and will be monitored for 2 h (visit (V) 1). After 4 and 8 months, patients randomized in the “liberated diet group” will be allowed to increase the small amounts to 1/50 (V2) and 1/10 (V3) if the amounts consumed thus far were well tolerated. The first consumption of each next higher amount will be performed at the study center, and subjects will be monitored for at least 2 h under medical supervision. Products with precautionary allergen labelling can be consumed after the daily consumption of at least 100 mg peanut or tree nut protein is reached. To investigate compliance and tolerability of the dietary interventions and to promote retention, patients will be asked to maintain a weekly diary and are contacted by phone 2 weeks after each visit (phone call (PC) 1, PC2, and PC3).

The study intervention will prematurely be discontinued for a subject if further participation in the trial presents a health risk for the subject in the judgment of the investigators, i.e., pregnancy, objective allergic reaction to the study intervention “liberated diet” on 1/100 of the individual initial threshold dose, severe adverse event related to the study intervention “liberated diet” requiring intensive care treatment or non-compliance.

Peanut and/or tree nut-allergic patients randomized in the “complete allergen avoidance group” will receive educational advice including verbal and written instructions by a dietician/nutritionist on standard peanut and/or tree nut avoidance as in routine clinical practice [10]. This includes avoidance of products with precautionary allergen labelling.

All participants may continue their usual medications including those taken for any concomitant disease including asthma, allergic rhinitis, or atopic dermatitis throughout the study. However, usage of antihistamines must be discontinued 3–5 days before final OFC at month 12 (V4). As medically indicated in the case of diagnosis of peanut or tree nut allergy, all patients should carry their emergency treatment medicine (including an adrenaline auto injector) at all times throughout the time course of the study. Furthermore, participants will be instructed to contact the study site by phone in the case of any objective immediate-type allergic reactions occurring after food consumption including accidental allergic reactions to peanut, tree nut or other food allergens, or recurrent gastrointestinal (GI) symptoms.

### Exposure (Cohort study)

Peanut- and/or tree nut-tolerant patients will receive educational advice including verbal and written information by dieticians/nutritionists on standard peanut and/or tree nut introduction and regular consumption as in routine clinical practice. This includes the advice to consume peanuts and/or tree nuts or products containing these allergens at least 3 times per week. Participants will be phone called three times throughout the study to investigate compliance and tolerability of the advice (regular consumption of the food and any immediate-type allergic reactions to the food). Furthermore, participants will be instructed to contact the study site by phone in the case of any objective immediate-type allergic reactions occurring after food consumption including accidental allergic reactions to peanut, tree nut, other food allergens, or recurrent GI symptoms.

### Primary endpoint

#### Randomized controlled trial (RCT)

The primary endpoint is the clinical tolerance to peanut or tree nuts after 1 year assessed by OFC (up to the repetitive cumulative dose).

#### Cohort study

The primary endpoint is the maintenance of clinical tolerance to peanut and/or tree nuts after 1 year assessed by feeding of the former tolerated cumulative dose.

Clinical tolerance is defined as the absence of allergic symptoms other than subjective symptoms or mild erythema, oral pruritus, mild nausea, mild abdominal pain, mild rhinitis, and/or less than three perioral hives during OFC or supervised feeding of the repetitive cumulative peanut and/or tree nut dose according to PRACTALL guidelines [32].

### Secondary endpoints

#### Randomized controlled trial

- Clinical tolerance to peanut after 1 year assessed by OFC (up to the repetitive cumulative dose) in % of peanut-allergic patients.
- Clinical tolerance to tree nuts after 1 year assessed by OFC (up to the repetitive cumulative dose) in % of tree nut-allergic patients.
- Clinical tolerance to individual type of tree nut after 1 year assessed by OFC (up to the repetitive cumulative dose) in % of the respective type of tree nut-allergic patients.
- Change in threshold level upon oral peanut or tree nut challenge after 1 year.
- Change in peanut- and/or tree nut-specific wheal size as well as IgE and IgG4 at 12 months from baseline.
- Incidence, frequency, severity, and relatedness of (serious) adverse events, especially immediate-type allergic reactions, GI problems, and accidental food exposure during the study assessed by diaries, telephone interviews, questionnaires, and during clinical visits.
- In participants with atopic dermatitis: change of severity of atopic dermatitis measured by SCORAD (Scoring atopic dermatitis) and EASI (Eczema Area and Severity Index) and topical eczema treatment.
- Use of epinephrine as a rescue medication during the study.
- Frequency of premature discontinuation of intervention due to adverse events.
- Change in generic and disease-specific quality of life (PedsQL - Pediatric Quality of Life Inventory, EQ-5D - European Quality of Life 5 Dimensions, FAQLQ - Food Allergy Quality of Life Questionnaire, FAIM - Food Allergy Independent Measure).
- Eating habits assessed by questionnaires.
- Burden of dietary behavior (assessed by a visual analog scale).

#### Cohort study

- Maintenance of clinical tolerance to peanut after 1 year assessed by feeding of the former tolerated cumulative dose in % of peanut-allergic patients.
- Maintenance of clinical tolerance to tree nuts after 1 year assessed by feeding of the former tolerated cumulative dose in % of tree nut-allergic patients.
- Maintenance of clinical tolerance to individual tree nuts after 1 year assessed by feeding of the former tolerated cumulative dose in % of the respective type of tree nut-allergic patients.
- Change in peanut- and/or tree nut-specific wheal size as well as IgE and IgG4 at 12 months from baseline.
- Dietary compliance (frequency and amount of peanut and/or tree nut consumption) during the study, assessed by questionnaires, telephone interviews, and during clinical visits.
- Frequency of allergic reactions to the food (peanut and/or tree nut) that was advised to consume regularly or to other food allergens during the observational period assessed by questionnaires during telephone interviews and clinical visits.
- In participants with atopic dermatitis: change of severity of atopic dermatitis (SCORAD, EASI) and topical eczema treatment at 12 months from baseline.
- Use of epinephrine as a rescue medication during the study.
- Change in generic and disease-specific quality of life during and after the study from baseline assessed by questionnaires (PedsQL, EQ-5D, FAQLQ, FAIM).
- Eating habits assessed by questionnaires at baseline compared to during and after the study.
- Burden of dietary behavior at two time points during the intervention assessed by a visual analog scale.

Non-compliance of the liberated diet group is defined as a repeated event of insufficient consumption of small allergen amounts, i.e., < 2 times per week for three weeks (either during 3 consecutive weeks or three repeated events of 1 week). Non-compliance of the strict avoidance group is defined as a repeated event of intended consumption of the specific allergen, i.e., > 3 times during the study period.

### Randomization and allocation concealment

Enrolled peanut- or tree nut-allergic patients will be randomized in a 1:1 ratio into one of two dietary intervention groups (liberated diet or complete allergen avoidance). Randomization will be stratified for age (1–5, 6–17, 18–67 years of age), allergen (peanut, tree nut), and threshold level at initial OFC (5th–6th, 7th–repetitive cumulative dose). The randomization list is computer-generated (SAS version 9.4, SAS Institute Inc., Cary, NC, USA) by the trial statistician and implemented within the REDCap database system by the data manager. Allocation of patients will be done by authorized study personnel using REDCap (Research Electronic Data Capture). Allocation concealment is assured as the result of the randomization will only be revealed after a patient is enrolled into the trial.

### Study procedures

Children and adults with a suspected or known primary peanut and/or tree nut allergy planning to undergo an oral peanut and/or tree nut (re-)challenge will be informed about the study. Before any study-specific procedure, written informed consent will be obtained from the subject or the subject’s parents/legal guardian according to participant’s age.

During the screening visit, the in- and exclusion criteria will be checked. Information on demographics, subject/household characteristics, relevant medical and allergy history, concomitant medication, dietary (food allergen exposure) history will be recorded. In addition, anthropometric measurements and a physical examination will be performed. In the case of eczema, severity will be assessed by SCORAD [33] and EASI score [34]. The investigators who assess the eczema status will be trained before delegation to the study team. A skin prick test will be performed with peanut (using roasted peanut and peanut extract), hazelnut (using raw and roasted hazelnut), cashew nut (using raw cashew nut), and walnut (using raw walnut) as well as a positive (1% histamine) and negative (0,9% NaCl) control (all extracts: ALK-Abelló, Germany). In addition, in case of suspected or diagnosed allergic rhinoconjunctivitis and/or allergic asthma, a skin prick test will be performed with aeroallergens (birch, mugwort, timothy, house dust mite, cat epithelium) at screening to assess baseline sensitization to aeroallergens. Study staff performing skin prick test will be trained before delegation to the study team. Blood, skin swabs (using DNA/ RNA Shield; Zymo Research, Irvine, CA, USA), stool (using OMNIgene-GUT tubes, OMR-200; DNA Genotek, Ontario, Canada), bed and living room house dust (using dustream® collector DU-ST-1; Indoor Biotechnologies LTD, Cardiff, UK), and saliva (using SalivaBio Swab; Suffolk, Great Britain) samples will be collected for mechanistic subprojects. Standard operating procedures are in place for biosample collection and study staff will be trained before delegation to the study team. Patients will be instructed how to collect stool and dust samples and written instructions will be given. Transepidermal water loss (TEWL) will be measured three times in a row (Tewameter TM 300 or Tewameter TM Hex, Courage + Khazaka electronic GmbH, Germany). Study staff assessing TEWL will be trained before delegated to the study. Eligible participants will undergo their planned OFC with peanut and/or tree nuts for clinical purpose. Vital sign measurement (temperature, pulse, blood pressure, oxygen saturation) will be performed prior to each food challenge. The food challenge, preferably double-blinded, placebo-controlled, are performed in accordance with clinical routine practice of the Charité-Universitätsmedizin Berlin, based on PRACTALL international guidelines for OFCs and stopped using standardized stopping criteria based on PRACTALL guidelines [32]. Roasted, defatted peanut flour, defatted hazelnut flour, crushed cashew, or walnuts (from store-bought whole nuts) will be used for OFC. In case of absence of any objective, allergic symptoms up to seven increasing dose steps at 30-min intervals using a semi log scale ranging approximately from 3 mg to 3 g food protein (depending on the individual allergen) followed by a cumulative dose up to 4.5 g food protein on another day will be administered. Dose steps and the corresponding allergen amounts used for OFCs with peanut or individual tree nuts are displayed in Appendix of the full protocol. Severity of objective immediate-type allergic reactions will be scored according to the grading system for food-induced anaphylaxis published by Sampson [35]. Depending on the outcome of the food challenge, subjects will be included in the TINA study as follows:

- Subjects with a positive oral peanut and/or tree nut challenge but who are able to tolerate at least the 5th challenge dose of peanut and/or tree nut will be enrolled into the TINA RCT (part I).
- Subjects with a negative oral peanut and/or tree nut challenge (successfully consuming and tolerating the repetitive cumulative dose of peanut and/or tree nut) will be enrolled into the TINA cohort study (part II).
- Subjects with a positive oral peanut and/or tree nut challenge reacting below the 5th dose will be considered as screening failures and will not be enrolled in the study.

#### Randomized controlled trial

At visit 1 (V1) patients with peanut and/or tree nut allergy eligible for the RCT will be randomized into two groups (Fig. 1) “liberated diet group” or “complete allergen avoidance group.” Patients following the liberated diet will get their first peanut or tree nut meal under medical supervision at the study center. They will be instructed to contact the study site by phone in the case of any objective immediate-type allergic reactions to food (either to the dietary regime or due to exposure to (other) food allergens) or recurrent GI symptoms. Patients will be asked to maintain a diary from V1 onwards and are called 2 weeks after V1 (PC1) to investigate compliance and tolerability of the dietary regime and to promote retention. Patients are required to visit the study site after 4 months (V2), 8 months (V3) and 12 months (V4) (Fig. 1, Table 1). At V2, V3, and V4, change of medical and allergy history, concomitant medication, family, and household characteristics will be recorded. In addition, anthropometric measurements and a physical examination will be performed including, in the case of eczema, its severity by SCORAD and EASI score. At V2 and V3 blood (adults only), stool, dust, and saliva samples will be collected and TEWL will be measured. Furthermore, patients randomized in the “liberated diet group” will be allowed to increase the small peanut or tree nut meal if the amounts consumed thus far were well tolerated. The first consumption of the next higher amount will be performed at the study center under medical supervision. Two weeks after V2 and V3, patients will be called (PC2 and PC3) to investigate compliance and tolerability and to promote retention. At V4, another skin prick test and oral challenge to peanut and/or tree nut will be performed (as described above), and blood, skin swab, stool, dust, and saliva samples will be collected.

**Table 1:**
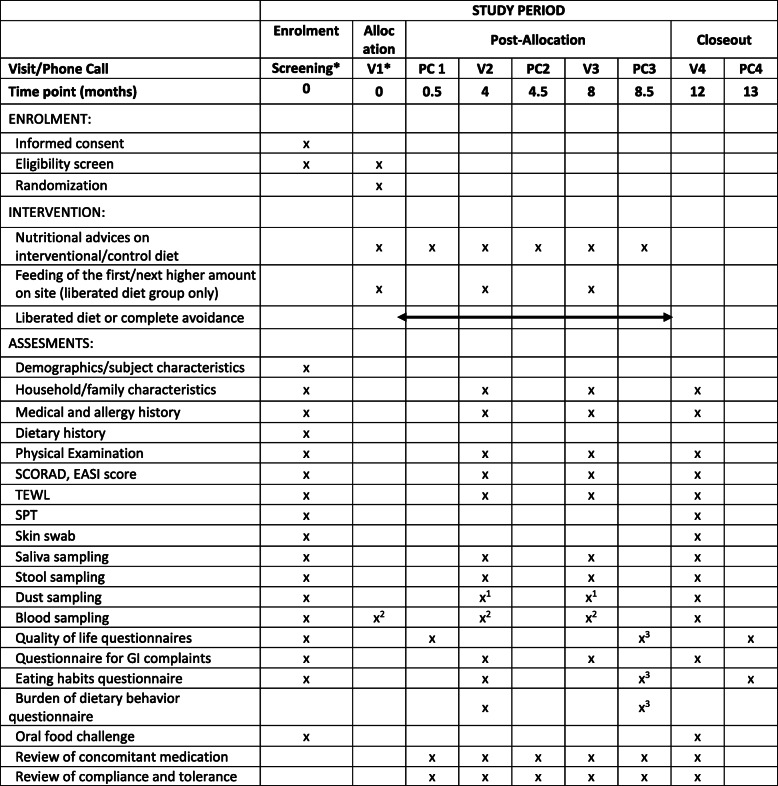
SPIRIT (Standard Protocol Items: Recommendations for Interventional Trials) figure showing important events and their respective time points during the study period in the randomized controlled trial (RCT)

Abbreviations: *V* Visit, *PC* Phone call, *SCORAD* Scoring atopic dermatitis, *EASI* Eczema area and severity index, *TEWL* Transepidermal water loss measurement, *SPT* Skin prick testing, *GI* Gastrointestinal, *I-C* Intervention-consumption group

* = Screening period can last up to 14 days and V1 might be on the last day of the screening period. The first day of the oral food challenge is defined as the first day of the screening period

1 = bed only

2 = adults only

3 = anytime between PC3 and V4

Generic and disease-specific quality of life questionnaires (PedsQL, EQ-5D, FAQLQ, and FAIM according to age) will be conducted before and 2 weeks after V1, anytime between PC3 and V4, as well as 4 weeks after V4 (remainder at PC4). The FAQLQ is a self-reported, food allergy-specific instrument intended to assess patients’ health-related quality of life and validated for different age groups [36–39]. FAIM is a food allergy independent measure reflecting the patient’s perceived food allergy severity and food allergy-related risk [40]. The validated generic quality of life questionnaires PedsQL and EQ-5D (assessed according to age) are assessed to compare the influence of food allergy with other various diseases on health-related quality of life [41, 42]. Eating habits will be assessed by questionnaire at screening, V2, anytime between PC3 and V4, as well as 4 weeks after V4. Burden of dietary behavior will be assessed using a visual analog scale at two time points during the intervention (V2 and between PC3 and V4). To assess possible GI complaints at screening, V2, V3, and V4, as well as in case of reported recurrent GI symptoms, the PEESS (Pediatric Eosinophilic Esophagitis Symptom Score) questionnaire will be filled out [43].

In case of discontinuation or deviation from the intervention protocol, all participants are encouraged to maintain the scheduled visits or phone calls and collection of all possible data is planned.

#### Cohort study

At V1, eligible subjects with challenge-proven tolerance to peanut and/or tree nut will be included in the cohort study (Fig. 1). Participants will be instructed to contact the study site by phone in the case of any objective immediate-type allergic reactions to food or recurrent GI symptoms. Participants will be contacted by phone 2 weeks, 4 months, and 8 months after V1 (PC1, PC2, PC3) to investigate compliance and tolerability concerning introduction and regular consumption of peanut and/or tree nut using a standardized questionnaire (Fig. 1, Table 2). Participants are required to visit the study site after 12 months (V4). At V4, change in medical and allergy history, concomitant medication, family and household characteristics will be recorded, compliance and tolerability will be assessed using a standardized questionnaire, anthropometric measurements and a physical examination will be performed including, in the case of eczema, its severity by SCORAD and EASI score. In addition, blood, skin swabs, stool, dust, and saliva samples will be collected and TEWL will be measured. Furthermore, participants will be fed one serving of peanut and/or tree nut (corresponding to the repetitive cumulative peanut and/or tree nut dose tolerated at entry food challenge) under medical supervision.

**Table 2:** Adapted SPIRIT (Standard Protocol Items: Recommendations for Interventional Trials) figure showing important events and their respective time points during the study period in the cohort study

	Study period					
	Enrolment	Observation				
Visit/Phone Call	Screening including V1^**a**^	PC 1	PC2	PC3	V4	PC4
Time point (months)	0	0.5	4	8	12	13
ENROLMENT:						
Informed consent	x					
Eligibility screen	x					
ASSESMENTS:						
Demographics/subject characteristics	x					
Household/family characteristics	x				x	
Medical and allergy history	x				x	
Dietary history	x					
Physical Examination	x				x	
SCORAD, EASI score	x				x	
TEWL	x				x	
SPT	x				x	
Skin swab	x				x	
Saliva sampling	x				x	
Stool sampling	x				x	
Dust sampling	x				x	
Blood sampling	x				x	
Quality of life questionnaires	x	x		x^b^		x
Questionnaire for GI complaints	x		x	x	x	
Eating habits questionnaire	x			x^b^		
Dietary compliance questionnaire			x	x	x	
Burden of dietary behavior questionnaire		x		x^b^		
Oral food challenge	x				x^c^	
Review of concomitant medication		x	x	x	x	
Review of compliance and tolerance		x	x	x	x	

Abbreviations: *V* Visit, *PC* Phone call, *SCORAD* Scoring atopic dermatitis, *EASI* Eczema area and severity index, *TEWL* Transepidermal water loss measurement, *SPT* Skin prick testing, *GI* Gastointestinal

^a^ = Screening period including V1 can last up to 14 days. The first day of the oral food challenge is defined as the first day of the screening period

^b^ = anytime between PC3 and V4

^c^ = at V4 the OFC consist of one feeding of the cumulative dose only

Generic and disease-specific quality of life questionnaires (PedsQL, EQ-5D, FAQLQ, and FAIM according to age) will be conducted before and 2 weeks after V1, anytime between PC3 and V4, as well as 4 weeks after V4 (remainder at PC4). Eating habits will be assessed by questionnaire at screening and anytime between PC3 and V4. Burden of dietary behavior will be assessed using a visual analog scale at two time points during the intervention (V2 and between PC3 and V4). To assess possible GI complaints at screening, PC2, anytime between PC3 and V4, as well as in case of reported recurrent GI symptoms, the PEESS questionnaire will be filled out.

### Data collection and management

REDCap (Research Electronic Data Capture) will be used as electronic case report form (eCRF) to collect and manage the study data. REDCap is hosted at the Charité – Universitätsmedizin Berlin and provides an intuitive interface for data entry for clinicians. In addition, it provides a data integration platform for the data from the mechanistic subprojects. Audit-trails will be integrated for tracking data entries, corrections, and import/export procedures. A unique participant number will be assigned to every screened patient consisting with a prefix and a consecutive numbering. This unique code will identify all patient-specific data, e.g., clinical study data as well as biosample results. SAEs will be documented in the REDCap eCRF and on paper documents. Data access and storage will follow the data security concept of the Charité – Universitätsmedizin Berlin including password-protected access to all computers and folders, which contain sensitive data. Pseudonymized data will be made available to all participating partners of the CRU. Study data will be retained up to 15 years after study completion and publication of results.

### Sample size calculation

#### Randomized controlled trial

The sample size calculation is based on the difference in the primary endpoint (clinical tolerance to peanut or tree nuts after 1 year) between the two treatment groups (liberated diet versus complete allergen avoidance). Analyzing 48 patients per group (96 patients in total) would result in over 90% power to detect a difference in tolerance development of 30% (liberated diet group) vs. 5% (complete allergen avoidance group) after 12 months, based on a two-sided chi-squared test with significance level 5%. These estimates are based on data on natural clinical tolerance development with about 20% of peanut-allergic patients and about 10% of tree nut-allergic patients developing oral tolerance later in life [6, 7, 44], and after 1 year of strict avoidance diet about 5% of peanut-allergic patients show natural tolerance development [18]. To compensate for a potential dropout rate of at most 20%, 60 patients will be randomized into each group (120 in total).

#### Observational cohort

The cohort study is considered exploratory. No formal sample size calculation has been performed. A number of 120 subjects to be included were considered realistic.

### Planned analysis

#### Randomized controlled trial

The primary endpoint is the clinical tolerance to peanut or tree nuts after 1 year assessed by OFC (up to the repetitive cumulative dose). The primary analysis of the primary endpoint will be performed by logistic regression with (fixed) factor treatment group and the stratification variables age (1–5 years, 6–17 years, 18–67 years), allergen (peanut, tree nuts), and threshold level (5th–6th dose, 7th–repetitive cumulative dose) as fixed factors. From this model, an adjusted odds ratio will be calculated with 95% confidence interval and a *p* value for the treatment group comparison. The analysis will be performed on the full analysis set (FAS) of patients analyzed according to their randomization group without imputation of missing values. The significance level will be set to 0.05 (two-sided). All other analyses will be considered explorative.

Several explorative sensitivity analyses will be performed for the primary endpoint:

1. A re-run of the primary analysis model with the per-protocol (PP) population (instead of the FAS) will be performed.
2. In case of relevant differences between the treatment groups with respect to baseline variables, the primary analysis will be repeated with further adjustment variables.
3. Subgroup analyses will be performed regarding the following subgroups:

• Age (1–5 years, 6–17 years, 18–67 years)

• Allergen (peanut, tree nuts)

• Threshold level (5th–6th dose, 7th–cumulative dose)

• Severity grade of allergic reaction at initial OFC

• Peanut or tree nut sIgE at screening

The explorative analysis of secondary endpoints will follow the same principle as the primary analysis of the primary endpoint, i.e., models with (fixed) factor treatment group and the stratification variables age (1–5 years, 6–17 years, 18–67 years), allergen (peanut, tree nuts), and threshold level (5th–6th dose, 7th–repetitive cumulative dose) factors. Continuous outcomes will be analyzed by analysis of covariance (ANCOVA) with the respective baseline value as additional covariate. Secondary endpoints will generally be analyzed with the FAS; selected secondary endpoints will also be analyzed with the PP population.

Concerning safety endpoints, the nature, frequency, and severity of adverse events and safety variables, including serious adverse events, will be summarized descriptively by dietary intervention group.

As a general analysis strategy, missing data will not be imputed. No interim analyses for efficacy are planned. Further details will be described in the Statistical Analysis Plan (SAP), which will be finalized prior to data analysis.

#### Cohort study

The primary endpoint (percentage of subjects staying peanut tolerant) will be reported as absolute number and percentage with the corresponding 95% confidence interval. Other endpoints will be summarized descriptively. Explorative comparisons of endpoints vs. baseline values will be performed (with methods for paired data). Further details will be described in the SAP.

## Discussion

Primary peanut and tree nut allergy is rarely outgrown and, therefore, affects both children and adults. Up to now, strict allergen avoidance is recommended for patients with peanut and tree nut allergy, independently of their individual threshold level, whereas sensitized subjects with challenge-proven tolerance are advised to introduce the food in the diet and consume it on a regular basis. As a strict elimination, diet has a negative impact on the everyday life of patients, effective strategies to prevent persistent food allergy other than the current management recommendation of strict avoidance would be of major importance. This clinical trial, including 120 children and adults with challenge-proven peanut and/or tree nut allergy reacting at higher threshold doses (RCT, part I) and 120 sensitized subjects with tolerance to peanut and/or tree nut (cohort study, part II), will investigate whether a liberated diet compared to strict avoidance can prevent the persistence of peanut and tree nut allergy and whether regular consumption of peanut and/or tree nut prevents (re-)development of peanut and tree nut allergy.

To our knowledge, this is the first ongoing trial investigating a novel dietary management option with a liberated diet (consumption of sub-threshold allergen amounts using small servings of store-bought products containing peanut or tree nut) compared to strict avoidance for peanut- and tree nut-allergic children and adults with higher threshold levels. Next to our trial, the ongoing CAFETERIA study in the US investigates a similar approach in children with peanut allergy reacting only to higher amounts of peanut (registered on ClinicalTrails.gov, NCT03907397). This trial aims to include 98 children between 4 and 14 years of age with challenge-proven peanut allergy able to ingest at least 143 mg of peanut protein. Children are randomized either to ingestion of sub-threshold amounts of peanut in their diet depending upon their reaction threshold (using store-bought peanut butter) or standard care of strict avoidance.

Since many patients with peanut allergy have an allergy to tree nut in parallel, a strength of our study is the inclusion of patients with peanut and/or tree nut allergy. While in part I, the RCT, for practical and safety reasons, only one allergen (peanut, hazelnut, cashew or walnut) will be part of the intervention, in part II (the cohort study) we observe tolerant participants regarding all foods (peanut, hazelnut, cashew and walnut) they exhibited tolerance upon OFC. As primary peanut and tree nut allergy is rarely outgrown, a further strength is that we included patients of all age groups.

The results of the trial will be submitted for publication in peer-reviewed journals and will further be communicated to participants, health care providers, especially allergologists, as well as dieticians and nutritionists specialized in food allergy to further improve the management of peanut and tree nut allergy, as they may affect current dietary recommendations.

To summarize, the objective of this randomized trial is to investigate whether there is a better option than strict avoidance diet for the management of peanut and tree nut allergy. This clinical trial will help to determine the impact of allergen avoidance on natural tolerance development and whether the current dietary advice for peanut or tree nut-allergic patients with higher threshold levels is still valid. Our results would help to provide better and individualized recommendations for patients with peanut and tree nut allergy. Furthermore, in a large prospective observational cohort, we will assess compliance of the dietary recommendation regarding introduction and regular consumption of peanut and tree nut in challenge-proven tolerant children and adults and its impact on maintenance of tolerance.

## Trial status

This is an abridged protocol based on protocol version 5.0 dated 09 September 2021. The first patient was randomized in March 2020 and the last patient is planned to be enrolled in the third quarter 2022.

## Supplementary Information

**Additional file 1.** The SPIRIT Checklist.

**Additional file 2.** Information Sheet (in German).

**Additional file 3.** Consent Form (in German).

## Data Availability

All data generated in the CRU will be curated and organized into a set of files, shared on an online, publicly available data repository after peer-reviewed publication (preservation and accessibility of the data) to ensure potential secondary analyses, long-term archiving, and reuse by other researchers (scientific recognition). Besides the study protocol, publications are planned for the results in peer-reviewed journals. In addition, results will be communicated in lay language to participants and health care providers as they may affect current dietary recommendations for patient with peanut and or tree nut allergy.

## Abbreviations

ANCOVA: Analysis of covariance
BIH: Berlin Institute of Health
CRF: Case Report Form
CRU: Clinical research unit
DBPCFC: Double-blind Placebo-Controlled Food Challenge
DFG: German Research Foundation
EASI Score: Eczema Area and Severity Index Score
eCRF: Electronic Case Report Form
EMA: European Medicines Agency
EQ-5D: European Quality of Life 5 Dimensions
FAIM: Food allergy independent measure
FAQLQ: Food Allergy Quality of Life Questionnaire
FAS: Full Analysis Set
FDA: Food and Drug Administration
IgE: Immunoglobulin E
IgG4: Immunoglobulin G4
LEAP trial: Learning Early About Peanut trial
OFC: Oral Food Challenge
PC: Phone Call
PedsQL: Pediatric Quality of Life Inventory
PEESS: Pediatric Eosinophilic Esophagitis Symptom Score
PN: Peanut
PP: Per-Protocol
PRACTALL: PRACTical issus of ALLergology, Joint United States/European Union Initiative
REC: Research Ethics Committee
REDCap: Research Electronic Data Capture
NaCl: Sodium chloride
SAE: Serious adverse event
SAP: Statistical Analysis Plan
SCORAD: SCORing Atopic Dermatits
SPT: Skin Prick Test
SPIRIT: Standard Protocol Items: Recommendations for Interventional Trials
TEWL: TransEpidermal Water Loss
TINA: Tolerance Induction through Non-avoidance to prevent persistent food Allergy
TN: Tree Nut
UK: United Kingdom
V: Visit
