# Supplementary material for: Tolerance induction through non-avoidance to prevent persistent food allergy (TINA) in children and adults with peanut or tree nut allergy: rationale, study design and methods of a randomized controlled trial and observational cohort study
Source: Trials. 2022 Mar 28;23:236. doi: 10.1186/s13063-022-06149-4 (PMC8962184; doi:10.1186/s13063-022-06149-4)
Supplement: Supplementary file 2 — Additional file 2. Information Sheet (in German). [file 13063_2022_6149_MOESM2_ESM.pdf]

CHARITÉ

ChariteCentrum für Innere Medizin und Dermatologie

**KLINIK FÜR DERMATOLOGIE, VENEROLOGIE UND ALLERGOLOGIE**
Klinikdirektor: Prof. Dr. med. K. Ghoreschi
Charitéplatz 1 | 10117 Berlin

Klinik mit zertifiziertem Qualitätsmanagementsystem nach DIN EN ISO 9001:2008

**Allergologie und Immunologie**
**Leiterin: Prof. Dr. med. M. Worm**

Kontakt Studienteam
Tel.: +49 30 450 518 003, -305 und -417
Fax: +49 30 450 7518968
E-Mail: acc-studien@charite.de

www.derma.charite.de
www.allergie-centrum-charite.de

Information sheet for adults

# Studieninformation

für die Teilnahme an der Studie

## Förderung der Toleranzentwicklung durch „Nicht-Vermeidung" zur Verhinderung einer persistierenden Nahrungsmittelallergie

**Sehr geehrte Studieninteressentin, sehr geehrter Studieninteressent,**

**Da Sie eine bekannte Erdnuss- und/oder Schalenfruchtallergie („Nüsse") haben oder ein Verdacht darauf besteht, möchten wir Sie einladen an unserer Studie teilzunehmen, die Ihre Ernährung auf die Toleranzentwicklung untersucht!**

**Diese Information soll Sie über die Teilnahme und den Ablauf der Studie aufklären. Sollten Sie Fragen zur Studie haben oder weitere Informationen wünschen, können Sie sich jederzeit an uns wenden.**

### Was sind Nahrungsmittelallergien?

In Industrieländern sind bis zu 8% der Kinder und 5% der Erwachsenen von einer Nahrungsmittelallergie betroffen. Erdnüsse und Schalenfrüchte gehören dabei zu den häufigsten Auslösern. Zu den Schalenfrüchten („Nüssen") zählt man die Haselnuss, Walnuss, Mandel, Cashewkerne, Pekannuss, Paranuss, Macadamianuss und Pistazie. Allergische Reaktionen auf Nahrungsmittel können sich auf unterschiedliche Weise äußern: Hautausschlag, Nesselsucht, Bauchschmerzen, Erbrechen, Durchfall, Atembeschwerden oder Kreislaufprobleme. Insbesondere Erdnuss und Schalenfrüchte können zu schweren allergischen Reaktionen führen.

### Was bedeutet Toleranzentwicklung?

Viele Kinder mit einer Nahrungsmittelallergie verlieren Ihre Allergie über die Zeit (Toleranzentwicklung), während andere sie bis ins Jugendlichen- oder Erwachsenenalter behalten. Die meisten Kinder mit einer Hühnerei- oder Kuhmilchallergie können das Nahrungsmittel nach 1-2 Jahren wieder problemlos essen. Dagegen verlieren nur ca. 10-20% der Betroffenen Ihre Erdnuss- oder Schalenfruchtallergie. Wir verstehen bis heute nicht, warum es diesen Unterschied zwischen den verschiedenen Allergenen gibt. Wir vermuten aber, dass ein Grund für die unterschiedliche Toleranzentwicklung der unbewusste, regelmäßige Verzehr sehr geringer Mengen   Kuhmilch und Hühnerei in der alltäglichen Ernährung sein könnte, da diese Allergene als Grundnahrungsmittel sehr häufig in Lebensmitteln verarbeitet werden. Für Erdnuss und Schalenfrüchte scheint dies unwahrscheinlich.

### Derzeitige Ernährungsempfehlungen

**Patient*innen mit einer Allergie** gegen Erdnuss oder Schalenfrüchte wird empfohlen, das Allergen strikt in Ihrer Ernährung zu meiden. Diese Empfehlung gilt für alle Patient*innen, unabhängig davon bei welcher Menge des Allergens es zu der allergischen Reaktion kam (individueller Schwellenwert), also z.B. bereits bei einem kleinen Krümel Nuss oder erst bei zehn Nüssen. Diese strenge Eliminationsdiät schränkt die Betroffenen im Alltag sehr ein. Wir vermuten, dass

diese strenge Eliminationsdiät, wie sie derzeit für alle Patient*innen empfohlen wird, der natürlichen Toleranzentwicklung möglicherweise entgegenwirkt.

Dagegen sollen **tolerante Personen**, die keine Symptome nach dem Verzehr von Erdnuss oder Schalenfrüchten zeigen, das Nahrungsmittel regelmäßig verzehren, um das Wiederauftreten der Allergie zu verhindern.

## Zweck der Studie

Ziel dieser Studie ist es zu untersuchen

**I** welchen Einfluss eine strenge Eliminationsdiät im Vergleich zu einer gelockerten Eliminationsdiät (also ein regelmäßiger Verzehr geringer Allergenmengen, unterhalb des individuellen Schwellenwerts) auf die natürliche Toleranzentwicklung **bei Patient*innen mit einer Erdnuss- und/oder Schalenfruchtallergie** hat.

**II** Parallel möchten wir beobachten, in welchem Maße der reguläre Allergenverzehr **bei toleranten Personen** das Wiederauftreten der Allergie verhindern kann.

Außerdem werden wir untersuchen, welche Mechanismen im Körper bei der Toleranzentwicklung eine Rolle spielen. Die Ergebnisse dieses Projekts können sehr hilfreich sein, um in Zukunft neue Ernährungsempfehlungen für Patient*innen mit einer Nahrungsmittelallergie aussprechen und diese somit behandeln zu können. Insgesamt sollen (I) 120 Kinder und Erwachsene mit einer Erdnuss- und/oder Schalenfruchtallergie und (II) 120 mit einer Toleranz auf Erdnuss und/oder Schalenfrüchte in die Studie eingeschlossen werden.

Die Studie ist Teil einer interdisziplinären klinischen Forschungsgruppe „Food Allergy and Tolerance - FOOD@“. Dabei handelt es sich um einen Zusammenschluss führender Institutionen der Charité - Universitätsmedizin Berlin, um die zugrundeliegenden Mechanismen bei der Entstehung und der Toleranzentwicklung von Nahrungsmittelallergien bei Kindern und Erwachsenen zu untersuchen.

## Wer kann teilnehmen?

Kinder ab 12 Monaten sowie Erwachsene bis 67 Jahren können an der Studie teilnehmen, wenn:

- der Verdacht auf eine Erdnuss- und/oder Schalenfruchtallergie besteht und daher eine orale Nahrungsmittelprovokation in unserer Klinik geplant ist.
- Sie eine bekannt Erdnuss- und/oder Schalenfruchtallergie haben und eine Re-Provokation durchgeführt werden soll, um zu untersuchen, ob eine Toleranzentwicklung stattgefunden hat.

Falls sich bei der Provokation herausstellt, dass eine Toleranz vorliegt, können Sie teilnehmen. Stellt sich bei der Provokation heraus, dass eine Allergie besteht, können Sie ebenfalls teilnehmen, sofern Symptome erst ab einer bestimmten Gabe der Provokation auftraten (ab etwa einer Nuss).

**Ihre Teilnahme ist freiwillig.** Nur wenn Sie zustimmen, können Sie in die Studie einbezogen werden. Wenn Sie nicht teilnehmen möchten, beeinträchtigt dies nicht Ihre weitere medizinische Behandlung. Sie haben keine Nachteile zu befürchten. Durch Ihre Teilnahme an der Studie entstehen Ihnen keine Kosten.

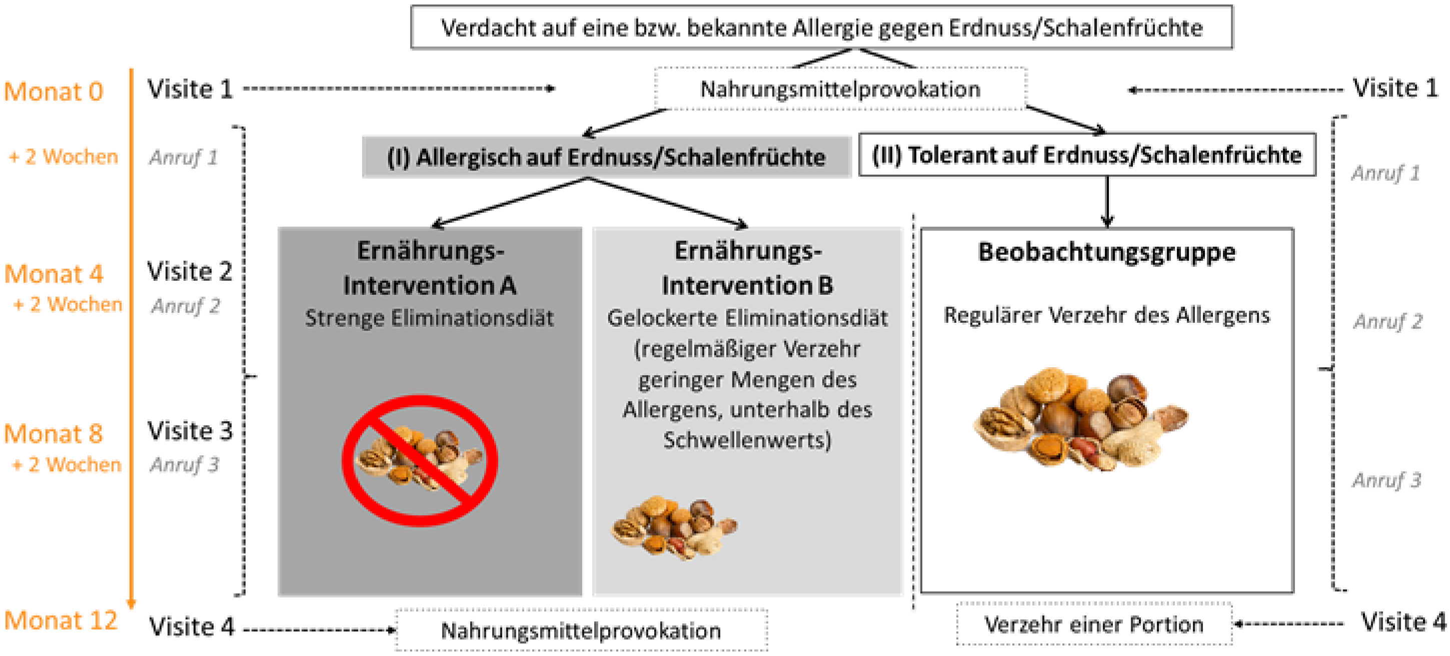

**Abbildung 1: Studiendiagramm**

## Was genau passiert in der Studie?

Je nachdem ob Sie im Rahmen der Nahrungsmittelprovokation durch das ärztliche Fachpersonal als allergisch oder tolerant beurteilt werden und alle sonstigen Ein- und Ausschlusskriterien erfüllt sind, werden Sie in eine der beiden Studienarme eingeschlossen (Abbildung 1). Die Studie begleitet Sie über 1 Jahr.

**I Allergisch auf Erdnuss und/oder Schalenfrüchte**
**→ Ernährungs-Interventionsgruppen**

Per Zufallsprinzip werden Sie entweder der Gruppe A zugeordnet, die Erdnuss und/oder Schalenfrüchte streng meidet, oder der Gruppe B, die kleine Mengen Erdnuss und/oder Schalenfrüchte regelmäßig verzehrt (siehe Abbildung 1).

In der Gruppe A werden Sie von unseren Ernährungsfachkräften bezüglich einer standardmäßigen, <u>strengen Eliminationsdiät</u>, die auch das Meiden von Produkten mit „Spurenhinweisen" auf das entsprechende Allergen beinhaltet, beraten.

In der Gruppe B werden Sie bezüglich einer <u>gelockerte Eliminationsdiät</u> beraten. Dabei sollen geringe Mengen an Erdnuss und/oder Schalenfrüchte unterhalb des individuellen Schwellenwerts mindestens 3-mal pro Woche, vorzugsweise täglich, verzehrt werden. Begonnen wird dabei weit unterhalb des individuellen Schwellenwerts (1/100). Nach 4 und 8 Monaten, wird die erlaubte Verzehrsmenge jeweils erhöht. Sie erhalten von uns genaue Informationen welche Produkte in welcher Menge verzehrt werden dürfen. Sobald Sie eine bestimmte Menge des Allergens verzehren, dürfen auch Produkte mit „Spurenhinweis" auf das entsprechende Allergen verzehrt werden.

Nach der Eingangsuntersuchung (V1), die im Rahmen der Nahrungsmittelprovokation durchgeführt wird, werden Sie im Verlauf der Studie noch zu weiteren drei Besuchen an unser Studienzentrum kommen (V2, V3 und V4). Bei der Abschlussvisite (V4) erfolgt eine erneute Nahrungsmittelprovokation, um den Erfolg der Ernährungsintervention zu untersuchen, also ob sich bei Ihnen an der Reaktion auf Erdnuss und/oder Schalenfrüchte etwas geändert oder sich evtl. eine Toleranz entwickelt hat. Patient*innen mit einer Erdnussallergie und optional auch für Schalenfruchtallergiker*innen ist jeweils 7 Tage, 14, 21 und 28 Tage, nach der oralen Nahrungsmittelprovokation eine einzelne Blutentnahme im Studienzentrum vorgesehen.

**II Tolerant auf Erdnuss und/oder Schalenfrüchte**
**→ Beobachtungsgruppe**

Sie erhalten von unseren Ernährungsfachkräften eine Beratung, bezüglich einer <u>standardmäßigen Einführung von Erdnuss und/oder Schalenfrüchten</u> in Ihre Ernährung. Dies beinhaltet die Empfehlung Erdnuss und/oder Schalenfrüchte bzw. Produkte, die diese Nahrungsmittel enthalten, mindestens 3-mal pro Woche zu verzehren.

Nach der Eingangsuntersuchung (V1), die im Rahmen der Nahrungsmittelprovokation durchgeführt wird, werden Sie im Verlauf der Studie noch zu einem weiteren Besuch an unser Studienzentrum kommen (V4). Bei der Abschlussvisite (V4) werden Sie gebeten, die Menge des Nahrungsmittels unter ärztlicher Aufsicht zu verzehren, die Sie bereits bei der Nahrungsmittelprovokation zu Studieneinschluss vertragen hatte, um zu überprüfen, ob die Toleranz nach wie vor besteht. Studienteilnehmer*innen, die eine Toleranz auf Erdnüsse aufweisen (keine Beschwerden nach der Erdnussprovokation), optional auch Schalenfruchttolerante Studienteilnehmer*innen, ist jeweils 7, 14, 21 und 28 Tage, nach der oralen Nahrungsmittelprovokation, zu einer einzelnen Blutentnahme in das Studienzentrum zu kommen.

### Studienablauf

Alle Maßnahmen und Untersuchungen, die in der Studie durchgeführt werden, werden in den folgenden Abschnitten erklärt. Im Verlauf der Studie werden Sie dreimal telefonisch kontaktiert, um Ihre Ernährung und die Verträglichkeit zu erfassen (PC1, PC2, PC3). Hierfür werden Sie zusätzlich gebeten fortlaufend ein Tagebuch zu führen (nur Interventionsgruppen), sowie zu vier Zeitpunkten einen Fragebogen auszufüllen.

Zu Beginn, während und am Ende der Studie bitten wir Sie einen Fragebogen zur Lebensqualität und dem Ernährungsverhalten auszufüllen.

<u>Anamnese und körperliche Untersuchung</u>

Bei jeder Visite (V1-V4) führen wir eine Anamnese (Erhebung der Krankengeschichte) durch und werden Sie körperlich untersuchen. Besonderes Augenmerk werden wir dabei auf das Vorliegen und den Verlauf allergischer Erkrankungen und Ihrem Hautstatus in Bezug auf eine atopische Dermatitis legen. Hierfür werden wir den Wasserverlust der Haut (transepidermaler Wasserverlust TEWL) messen. Dieser kann als Maß für die Einschränkung der Hautbarrierefunktion herangezogen werden und wird auf der Hautoberfläche bestimmt. Es handelt sich dabei um eine nur wenige Sekunden andauernde, schmerz- und belastungsfreie Untersuchung, die wir bei jeder Visite durchführen werden.

Die Untersuchungen werden von allergologisch geschulten Ärzt*innen durchgeführt. Selbstverständlich besteht genügend Zeit um Ihre individuellen Fragen zu beantworten. Darüber hinaus werden wir Sie bitten einen Fragebogen zu Ihrem Gesundheitszustand auszufüllen.

Hautpricktest

Bei allen Teilnehmer*innen wird ein Hautpricktest durchgeführt. Mit diesem Hauttest können wir Ihren Sensibilisierungsstatus (Allergiebereitschaft z.B. gegenüber Nahrungsmitteln oder Pollen) bestimmen. Der Hautpricktest zählt zu den üblichen Untersuchungsmethoden in der medizinischen Praxis bei dem Verdacht auf Nahrungsmittelallergien oder zur Verlaufskontrolle bei einer bestehenden Nahrungsmittelallergie. Dabei werden verschiedene Nahrungsmittelallergene (z.Bsp Erdnuss) und ggf. inhalative Allergenen mit einer kleinen Nadel unter die Haut am Unterarm gebracht (praktisch schmerzlos). Nach 15 Minuten wird der Test abgelesen, wobei eine lokale Rötung und Quaddelbildung ein positives Testergebnis liefert.

Gewinnung von Blutproben

Die Blutentnahme gehört zu den üblichen Untersuchungsmethoden in der medizinischen Praxis bei dem Verdacht auf Nahrungsmittelallergien oder zur Verlaufskontrolle bei einer bestehenden Nahrungsmittelallergie. Ihnen wird zu Beginn (V1) und am Ende (V4) der Studie ca. 60 ml und im Verlauf der Studie (V2 und V3) ca. 10 ml Blut entnommen. Erfolgt im Rahmen der Visite eine Nahrungsmittelprovokation, können wir das Blut über den intravenösen Zugang entnehmen, der dabei routinemäßig gelegt wird, um im Notfall sofort Medikamente geben zu können. Bei den Visiten mit einer Nahrungsmittelprovokation wird Ihnen vor 10 ml) und nach der Provokation (ca. 20 ml) Blut abgenommen. Bei Studienteilnehmer*innen mit einer Erdnussallergie oder -toleranz (optional für Schalenfrucht) werden zusätzlich ca. 50 ml Blut vor, 7, 14, 21 und 28 Tage nach der Provokation abgenommen.

Das Blut wird verwendet, um wie in der Routinediagnostik festzustellen, ob verschiedene Allergieantikörper gegen Erdnuss und/oder Schalenfrüchte und ggf. weitere Nahrungsmittelallergene vorliegen. Das restliche Blut wird für weitere verschiedene Labortests verwendet. Hierbei sollen Zellen, Antikörper und Botenstoffe, sowie erblich bedingte (genetische) Faktoren untersucht werden, die mit der Entwicklung der Toleranz einhergehen und solche, die die Schwere einer allergischen Reaktion voraussagen können. Genetische Faktoren und Umweltfaktoren bestimmen gemeinsam das Risiko, eine allergische Erkrankung zu entwickeln, aber vermutlich auch auf die natürliche Toleranzentwicklung. Es ist wichtig herauszufinden, welche Gene daran an der Toleranzentwicklung beteiligt sind und welche Auswirkungen sie haben. Erbvarianten, die krankheitsrelevant sind, kommen bei Patient*innen häufiger vor, als bei Gesunden. Bei einer das ganze Erbgut umfassenden Untersuchung, der genomweiten Sequenzierung, können wir alle erblichen Varianten im Erbgut (Genom) identifizieren und prüfen, ob sie bei den Betroffenen besonders häufig vorkommen und welche Auswirkungen sie haben. Dabei werden die Daten von Hunderten bis Tausenden Patient*innen und Gesunden verglichen, sodass die Ergebnisse nur für die Gesamtgruppe und nicht für einzelne Patient*innen ausgewertet werden. Ziel ist es eine diagnostische Möglichkeit zu finden, um die Nahrungsmittelprovokation möglicherweise zukünftig umgehen zu können.

Alle Ergebnisse, die in der Routinediagnostik von Bedeutung sind, werden Ihnen selbstverständlich mitgeteilt und mit Ihnen besprochen.

Gewinnung von Hautabstrichen, Stuhl-, Speichel- und Hausstaubproben

Bei jeder Visite (V1-V4) werden Speichel-, Stuhl- und Hausstaubproben eingesammelt. Bei der Eingangs- und Abschlussvisite (V1 und V4) werden außerdem Hautabstriche durchgeführt. Die Hautabstriche und Speichelproben werden im Laufe der Visite gewonnen, die Stuhl- und Staubproben können von Ihnen zu Hause gewonnen und zur jeweiligen Visite mitgebracht werden.

Die Haut und der Darm sind mit vielen verschiedenen Bakterien besiedelt, die uns Menschen nicht schaden. In ihrer Gesamtheit nennen wir sie „Haut-“ oder „Darmflora“. Die Bakterienflora kann die Immunantwort des Menschen und speziell die Entwicklung allergischer Erkrankungen beeinflussen. Mit Hilfe der Stuhlproben und Hautabstriche, möchten wir die haut- und darmspezifische Bakterienflora und deren Einfluss auf die Toleranzentwicklung bei Nahrungsmittelallergien untersuchen. Hierfür wird ein Hautabstrich an zwei verschiedenen Stellen der Haut mit einem feuchten Wattebausch durchgeführt.

Der Stuhl wird mit Hilfe eines bereitgestellten Probenbehälters aus einem Auffangpapier gewonnen. Außerdem soll der Einfluss der Ernährung und weiterer Umweltfaktoren insbesondere der Besitz eines Hundes hierauf untersucht werden.

Wir wissen, dass Allergene im Hausstaub eine wichtige Rolle bei Nahrungsmittelallergien spielen. Daher bitten wir sie Hausstaubproben an bestimmten Orten Ihres Wohnraums mit einem speziellen Staubsaugeraufsatz zu sammeln.

Die Untersuchung von Speichel findet in verschiedenen Bereichen der Medizin bereits Anwendung. Wir wollen untersuchen, ob Speichel auch für die Allergiediagnostik geeignet ist und insbesondere eine Toleranzentwicklung voraussagen kann. Dies ist insbesondere interessant, da die Speichelgewinnung unkompliziert und schmerzfrei durchgeführt werden kann.

Hierfür werden wir mittels eines langen Watteträgers Speichel aus der Mundhöhle gewinnen.

Orale Nahrungsmittelprovokation
Auch die orale Nahrungsmittelprovokation ist ein Standardverfahren in der Routinediagnostik, um eine Verdachtsdiagnose auf Nahrungsmittelallergie zu bestätigen oder um bei bestätigter Nahrungsmittelallergie zu untersuchen, ob Sie im Laufe der Zeit tolerant geworden sind und das Nahrungsmittel möglicherweise wieder vertragen. Routinemäßig werden orale Nahrungsmittelprovokationen stationär durchgeführt.
Die Provokation wird in der Regel Placebo-kontrolliert und doppelblind durchgeführt. Dabei erhalten Sie aufsteigende Mengen des Nahrungsmittels, das im Verdacht steht, die Allergie auszulösen. Das verdächtige Nahrungsmittel wird in ein verträgliches Lebensmittel (z.B. Brei) untergemischt. Zum Vergleich gibt es aber auch Portionen, die das verdächtige Nahrungsmittel nicht enthalten (sogenanntes Placebo). Doppelblind bedeutete, dass weder Sie noch das anwesende ärztliche Fachpersonal beim Test wissen darf, in welcher Mahlzeit das „verdächtige" Nahrungsmittel enthalten ist. Die Durchführung pro Nahrungsmittel dauert zwei Tage, während und nach jeder Mahlzeit werden Sie sorgfältig überwacht, um zu sehen, ob allergieverdächtige Reaktionen auftreten. Der Nahrungsmittelprovokationstest wird gestoppt, wenn eine klinische Reaktion auf das provozierte Nahrungsmittel auftritt. Routinemäßig wird vor Beginn der Nahrungsmittelprovokation ein Zugang gelegt, um im Notfall sofort Medikamente geben zu können.

## Was geschieht mit den Bioproben die wir gewonnen haben?

Die im Rahmen der Studie entnommenen Bioproben werden pseudonymisiert (verschlüsselt) und in dieser Form an folgende Labore zu den oben genannten Untersuchungszwecken übermittelt. Den Forschern ist kein Rückschluss auf Ihre Person möglich. Die Bioproben werden nicht in den Laboren gelagert, sondern nach Analyse vernichtet:

- Pädiatrie m. S. Pneumologie und Immunologie mit Intensivmedizin, Charité - Universitätsmedizin Berlin
  Augustenburgerplatz 1, 13353 Berlin
- Institut für Mikrobiologie und Infektions-Immunologie; Charité - Universitätsmedizin Berlin
  Hindenburgdamm 30, 12203 Berlin
- Max-Delbrück-Centrum, Charité - Universitätsmedizin Berlin; Robert-Rössle-Str. 10, 13092 Berlin
- Experimental and Clinical Research Center des Max-Delbrück-Centrum und der Charité
  Lindenberger Weg 80, 13125 Berlin
- Berlin-Brandenburg Center für Regenerative Therapien (BCRT); Augustenburger Platz 1, 13353 Berlin
- Klinik für Dermatologie, Venerologie und Allergologie, Charité - Universitätsmedizin Berlin
  Charitéplatz 1, 10117 Berlin
- Thermo Fisher Scientific, Servicelabor,
  Munzinger Straße 7, 79111 Freiburg
- Labor Berlin, Sylter Str. 2, 13353 Berlin

Ein Teil der Bioproben wird über einen Zeitraum von 10 Jahren nach Abschluss der Studie und Veröffentlichung der Ergebnisse ebenfalls pseudonymisiert im Labor der Klinik für Dermatologie, Venerologie und Allergologie (Charitéplatz 1, 10117 Berlin) und im Labor der Pädiatrie m. S. Pneumologie und Immunologie mit Intensivmedizin (Augustenburgerplatz 1, 13353 Berlin) gelagert, für die (unter anderem auch genetische) Analysen, in weiteren Projekten, die aus neuen wissenschaftlichen Erkenntnissen der nächsten Jahre hervorgehen.
Zugang zu den Bioproben hat nur autorisiertes Studienpersonal.
Bei jeder Erhebung, Speicherung und Übermittlung von Daten aus Biomaterialien im Rahmen von Forschungsprojekten bestehen Vertraulichkeitsrisiken (z. B. die Möglichkeit, Sie zu identifizieren), insbesondere im Hinblick auf die Information zur Erbsubstanz. Diese Risiken lassen sich nicht völlig ausschließen und steigen, je mehr Daten miteinander verknüpft werden können. Die Studienleitung versichert Ihnen, alles nach dem Stand der Technik Mögliche zum Schutz der Privatsphäre zu tun und Bioproben nur an Projekte weiterzugeben, die ein geeignetes Datenschutzkonzept vorweisen können.

## Was geschieht mit den Daten, die wir erhoben haben?

Die Speicherung und Analyse der Daten (inklusive der epigenetischen Daten) erfolgt in Zusammenarbeit mit dem:

- Pädiatrie m. S. Pneumologie und Immunologie mit Intensivmedizin, Charité - Universitätsmedizin Berlin
  Augustenburgerplatz 1, 13353 Berlin
- Institut für Sozialmedizin, Epidemiologie und Gesundheitsökonomie, Charité - Universitätsmedizin Berlin
  Charitéplatz 1, 10117 Berlin
- Institut für Physiologie, Charité - Universitätsmedizin Berlin; Charitéplatz 1, 10117 Berlin
- Experimental and Clinical Research Center des Max-Delbrück-Centrum und der Charité
  Lindenberger Weg 80, 13125 Berlin

Alle Studiendaten werden nur pseudonymisiert (d.h. ein Rückschluss auf Sie ist dort nicht möglich) weiter-

gegeben. Personenbezogene Daten verbleiben ausschließlich im Studienzentrum der Klinik für Dermatologie, Venerologie und Allergologie der Charité. Studienrelevante Unterlagen werden 10 Jahre nach Abschluss der Studie und Veröffentlichung der Ergebnisse im Studienzentrum aufbewahrt und anschließend auf sichere Weise entsorgt. Die gesammelten Daten werden elektronisch archiviert. Zugang zu den Daten hat nur autorisiertes Studienpersonal.

### Was sind die Vorteile einer Teilnahme?

Die Studie wird von allergologisch erfahrenen Ärzt*innen und Ernährungsfachkräften durchgeführt, die Sie intensiv betreuen. Die klinisch indizierte Allergiediagnostik (Messung von Allergieantikörpern, Nahrungsmittelprovokation) hilft bei der Beurteilung bestehender oder neu hinzugekommener Allergien. Sie kann im Falle einer Erdnuss- und/oder Schalenfruchtallergie aber auch helfen zu beurteilen, ob Sie das entsprechende Nahrungsmittel wieder vertragen. Falls Sie eine bestehende Erdnuss- und/oder Schalenfruchtallergie haben, besteht die Möglichkeit, dass Sie mit der neuen Ernährungsempfehlung schneller eine Toleranz gegen Erdnuss bzw. Schalenfrüchte entwickeln.

### Was ist der Nutzen der Teilnahme und der Studie für die Allgemeinheit?

Die weitergehenden Untersuchungen, wie die Analyse des Mikrobioms des Darms und der Haut sowie die Immunantwort des Körpers auf Nahrungsmittelallergene, werden das Verständnis der Entstehung und den Verlauf von allergischen Erkrankungen und der Toleranzentwicklung verbessern. Sie können längerfristig zur Entwicklung verbesserter Möglichkeiten der Früherkennung und Behandlung von Nahrungsmittelallergien beitragen. Die aktuellen Ernährungsempfehlungen für Patient*innen könnten ggf. aktualisiert werden und Patient*innen mit einer persistenten Nahrungsmittelallergie müssten das Nahrungsmittel nicht mehr streng meiden, was die Lebensmittelauswahl erleichtern und zu einer verbesserten Lebensqualität führen würde.

### Was sind mögliche Risiken einer Teilnahme?

Sie könnten sich bei der Blutabnahme leicht unwohl fühlen. Zur Betäubung der Haut kann auf Wunsch eine anästhesierende Creme aufgetragen werden. An der Stelle der Blutentnahme kann ein leichter Bluterguss entstehen. Infektionen sind unter Standardhygienebedingungen extrem selten.
Bei dem Hautpricktest tritt häufig lokaler Juckreiz auf, allergische Allgemeinreaktionen sind extrem selten. Der Hautpricktest wird nur von allergologisch geschultem Personal und unter Überwachung durchgeführt.
Bei der Teilnahme an einem Nahrungsmittelprovokationstest kann es unter anderem zu folgenden allergischen Reaktionen kommen: Juckreiz, Quaddeln (Nesselsucht), Erbrechen, Durchfall, Husten, pfeifende Atmung oder Verschlechterung einer bestehenden atopischen Dermatitis („Neurodermitis“). Schwere Reaktionen wie Anaphylaxie (Atemnot oder schwere Herz-Kreislauf-Reaktionen) treten nur in seltenen Fällen auf. Alle Nahrungsmittelprovokationstests werden von einem geschulten ärztlichen Fachpersonal streng überwacht, sodass allergische Reaktionen sofort behandelt werden können und eine Notfallversorgung ist zu jedem Zeitpunkt sichergestellt. Routinemäßig wird vor Beginn der Nahrungsmittelprovokation ein Zugang gelegt, um im Notfall sofort Medikamente geben zu können.
Bei der gelockerten Eliminationsdiät erwarten wir keine allergischen Symptome, da während der Provokation gezeigt werden konnte, dass Sie diese und weitaus höhere Mengen des Allergens vertragen. Zur Sicherheit wird die erste, sowie die zwei gesteigerten Verzehrsmengen des Allergens unter ärztlicher Aufsicht gegeben. Zwar können wir allergische Reaktionen nicht zu 100% ausschließen, schwere allergische Reaktionen sind jedoch sehr unwahrscheinlich.

### Was sind Umstände, die zum Abbruch der Studienteilnahme führen können?

Ihre Teilnahme an der Studie ist freiwillig und nur mit Ihrer schriftlichen Zustimmung auf der Einwilligungserklärung möglich. Sie haben jederzeit das Recht ohne Nennung von Gründen, die Teilnahme an der Studie zu beenden. Auch das studienärztliche Fachpersonal hat das Recht, Ihre Studienteilnahme aus Sicherheitsgründen, wegen Änderung der Maßnahmen oder aus anderen medizinischen Gründen zu beenden. Ein Abbruch beeinträchtigt in keiner Weise Ihre weitere medizinische Behandlung und Betreuung. Sie haben keine Nachteile zu befürchten.

### Wie werden meine Daten geschützt?

Im Rahmen der Studie werden personenbezogene Daten erhoben, verarbeitet und gegebenenfalls pseudonymisiert weitergegeben. Außerdem werden im Rahmen der Studie Bioproben (Blut, Speichel, Haut, Stuhl) erhoben und verarbeitet (siehe Abschnitt „Was geschieht mit den Bioproben die wir gewonnen haben?“). Die Verarbeitung der Daten erfolgt gemäß den einschlägigen rechtlichen Bestimmungen insbesondere der Datenschutzgrundverordnung (DSGVO),

dem Berliner Datenschutzgesetz (BerlDS) sowie §25 des Berliner Gesetz zur Regelung des Krankenhausrechts (LKG). Durch Ihre Unterschrift auf der Einwilligungserklärung erklären Sie sich damit einverstanden, dass das Studienteam von Prof. Dr. med. M. Worm Ihre personenbezogenen Daten zum Zweck der oben genannten Studie erheben und verarbeiten darf. Personenbezogene Daten sind z.B. Ihr Name, Geburtsdatum, Ihre Adresse und Daten zu Ihrer Gesundheit oder Erkrankung oder andere persönliche Daten, die während Ihrer Teilnahme an der Studie oder bei einer der Folgeuntersuchungen zweckgebunden erhoben wurden. Das Studienteam wird die personenbezogenen Daten ausschließlich für Zwecke der Verwaltung und Durchführung der Studie verwenden und diese, einem Pseudonym (verschlüsselte Codenummer) zugeordnet, für Zwecke der Forschung und statistischen Auswertung verwenden. Auf die Codenummer, die es erlaubt, die pseudonymisierten Daten mit Ihnen in Verbindung zu bringen, hat nur das Studienteam von Prof. Dr. med. M. Worm Zugriff. Dritte wie z.B. Sponsoren und deren befugte Mitarbeiter erhalten nur Einblick in personenbezogene Unterlagen, um die Qualität der Durchführung der Studie abzusichern. Bitte beachten Sie, dass die Ergebnisse der Studie in der medizinischen Fachliteratur veröffentlicht werden können, wobei Ihre Identität jedoch anonym bleibt, so dass die Veröffentlichung ohne personenbezogene Daten erfolgt. Für den Schritt der Anonymisierung erfragen wir Ihre Einwilligung, da es sich um eine Verarbeitung im Sinne von Art. 4 Absatz 2 DSGVO handelt. Da nach einer Anonymisierung ein Personenbezug nicht mehr herstellbar ist, ist der Anspruch auf die im folgenden aufgeführten Rechte (Auskunft, Berichtigung oder Löschung) nicht durchführbar.

Sie haben im Zusammenhang mit der Datenverarbeitung folgende Rechte:

Sie können jederzeit ihre erteilte Einwilligung widerrufen. Beachten Sie bitte, dass die bis dahin erfolgte Verarbeitung Ihrer Daten nicht berührt wird, Art. 7 Absatz 3 DSGVO.

Daneben haben Sie jeweils das Recht:

- auf Auskunft über alle Verarbeitungen der zur Person verarbeiteten und gespeicherten Daten und Empfänger, an die Daten weitergegeben werden oder wurden. Die Bereitstellung der Daten kann in einem maschinenlesbaren Format verlangt werden, Art. 15, 20 DSGVO.
- auf Berichtigung unrichtiger personenbezogener Daten, Art. 16 DSGVO.
- einer Weiterverarbeitung Ihrer personenbezogenen Daten zu widersprechen, die ohne Ihre Einwilligung aufgrund eines öffentlichen Interesses oder zur Wahrung berechtigter Interessen des Verantwortlichen erfolgt ist. Der Widerspruch einer Weiterverarbeitung ist zu begründen, so dass deutlich wird, dass besondere in Ihrer Person begründete Umstände das vorgenannte Interesse an einer Weiterverarbeitung überwiegt, Art. 21 DSGVO.
- auf Löschung/Vernichtung unter der Voraussetzung, dass bestimmte Gründe vorliegen. Dies ist insbesondere der Fall bei unrechtmäßiger Verarbeitung, wenn die Daten/Bioproben zu dem Zweck zu dem sie erhoben oder verarbeitet wurden nicht mehr notwendig sind, Sie die Einwilligung widerrufen und eine anderweitige Rechtsgrundlage für die Datenverarbeitung nicht gegeben ist oder anstelle des vorbenannten Widerspruchs nach Art. 21 DSGVO unter den dort genannten Voraussetzungen. Sofern die Löschung/Vernichtung die Ziele eines im wissenschaftlichen Interesse durchgeführten Forschungsprojektes zu Nichte machen oder wesentlich erschweren würde, besteht kein Recht auf Löschen/Vernichtung, Art. 17 Absatz III. Nach Ablauf einer Aufbewahrungszeit von 10 Jahren nach Beendigung der Studie und Veröffentlichung der Ergebnisse werden Ihre personenbezogenen Daten/Bioproben gelöscht/vernichtet.
- auf Einschränkung der Verarbeitung personenbezogener Daten, insbesondere wenn die Verarbeitung unrechtmäßig ist und Sie die Einschränkung anstelle des Löschens verlangen (siehe dort) oder solange streitig ist, ob die Verarbeitung personenbezogener Daten rechtmäßig erfolgt, Art. 18 DSGVO.

Zur Wahrnehmung der vorgenannten Rechte wenden Sie sich bitte an die für die Verarbeitung der personenbezogenen Daten verantwortliche Stelle:

Kontaktdaten Studienteam:
Charité - Universitätsmedizin Berlin
Klinik für Dermatologie, Venerologie und Allergologie
Prof. Dr. med. Margitta Worm
Dr. rer. medic. Sabine Dölle-Bierke
Charitéplatz 1, 10117 Berlin
E-Mail: acc-studien@charite.de

Bei Anliegen zur Datenverarbeitung und zur Einhaltung der datenschutzrechtlichen Anforderungen können Sie sich auch an folgende Stelle wenden:

Datenschutzbeauftragter:
Charité - Universitätsmedizin Berlin, Campus Mitte
Stabsstelle Datenschutz
Charitéplatz 1, 10117 Berlin
Tel.: 030 450 580016 | E-Mail: datenschutz@charite.de

Für den Fall, dass Sie eine Datenverarbeitung für rechtswidrig halten, haben Sie neben der Inanspruchnahme gerichtlicher Hilfe die Möglichkeit

Beschwerde einzureichen bei der für die Charité - Universitätsmedizin Berlin zuständigen Aufsichtsbehörde:

Berliner Beauftragte für Datenschutz und Informationsfreiheit
Friedrichstraße 219, 10969 Berlin
Tel.: 030 13889-0 | Fax: +49 30 2155050
E-Mail: mailbox@datenschutz-berlin.de

Die Daten-/Bioprobenerhebung zu wissenschaftlichen Zwecken ist kosten- und zeitintensiv, sodass die Verwendung bereits erhobener Daten/Bioproben dazu beitragen kann, beispielsweise neu entwickelte Analysemethoden schneller auf ihren potenziellen medizinischen Nutzen hin zu untersuchen. Daher erfragen wir in der Einwilligungserklärung Ihre Zustimmung zur Verarbeitung der Daten/Bioproben in konkreten Anschlussstudien bzw. um Zustimmung zu einer späteren Kontaktaufnahme zu dem Zweck, Ihre Einwilligung zur Verarbeitung der bereits erhobenen Daten/Bioproben oder zur Erhebung neuer Daten/Bioproben im Rahmen einer möglichen weiteren, zum gegenwärtigen Zeitpunkt noch nicht festgelegten Studie.

## Versicherungsschutz

Für diese Studie wurde keine spezielle Versicherung für die Patient*innen abgeschlossen. Die an der Studie beteiligten Mitarbeiter der Charité (Studienärzte und -ärztinnen, Ernährungsfachkräfte, Studienschwestern und -pfleger etc.) sind durch die Betriebshaftpflichtversicherung der Charité gegen Haftpflichtansprüche, welche aus ihrem schuldhaften Verhalten resultieren könnten, versichert.

## Wer finanziert und organisiert das Forschungsprojekt?

Diese Studie wird als Teil der klinischen Forschungsgruppe „Food Allergy and Tolerance - FOOD@“ durch Drittmittel der Deutsche Forschungsgesellschaft (DFG) finanziert.

## Haben Sie noch Fragen?

Wir hoffen, Ihnen alle Fragen zur Studie zufriedenstellend beantwortet zu haben. Bei Fragen oder Bedenken hinsichtlich Ihrer Studienteilnahme, insbesondere auch zu möglichen Risiken, oder wenn Sie zu irgendeinem Zeitpunkt mit bestimmten Punkten der Studie unzufrieden sind, können Sie sich jederzeit an uns wenden.

## Ihr Studienteam!

**Email:** acc-studien@charite.de
tina-studie@charite.de
**Tel:** 030-450518-003/-305/-417
Prof. Dr. med. Margitta Worm
Leiterin des Studienzentrums für Allergologie und Immunologie

**Wir danken Ihnen für Ihre Zeit und würden uns freuen, wenn Sie sich für die Teilnahme an unserem Forschungsprojekt entscheiden!**

CHARITÉ

CharitéCentrum für Frauen-, Kinder- und Jugendmedizin mit Perinatalzentrum und Humangenetik

**Klinik für Pädiatrie mit Schwerpunkt Pneumologie und Immunologie mit Intensivmedizin**
Klinikdirektor: Prof. Dr. med. Marcus Mall
Sektion Kinderallergologisches Studienzentrum
Leitung: Prof. Dr. med. Kirsten Beyer
Telefon Sekretariat: 030 – 450 566 037
Telefon Studienteam: 030 – 450 516 466
Fax: 030 – 450 566 931
E-Mail Studienteam: Kinder-allergiestudien@charite.de
www.charite-ppi.de

Information sheet for parents

# Studieninformation für Eltern

für die Teilnahme an der Studie

## Förderung der Toleranzentwicklung durch „Nicht-Vermeidung“ zur Verhinderung einer persistierenden Nahrungsmittelallergie (TINA-Studie)

**Sehr geehrte studieninteressierte Eltern,**

**Da Ihr Kind eine bekannte Erdnuss- und/oder Schalenfruchtallergie („Nüsse“) hat oder ein Verdacht darauf besteht, möchten wir Sie einladen an unserer Studie teilzunehmen, die die Ernährung Ihres Kindes auf die Toleranzentwicklung untersucht!**

**Diese Information soll Sie über die Teilnahme und den Ablauf der Studie aufklären. Sollten Sie Fragen zur Studie haben oder weitere Informationen wünschen, können Sie sich jederzeit an uns wenden.**

### Was sind Nahrungsmittelallergien?

In Industrieländern sind bis zu 8% der Kinder und 5% der Erwachsenen von einer Nahrungsmittelallergie betroffen. Erdnüsse und Schalenfrüchte gehören dabei zu den häufigsten Auslösern. Zu den Schalenfrüchten zählt man die Haselnuss, Walnuss, Mandel, Cashewkerne, Pekannuss, Paranuss, Macadamianuss und Pistazie. Allergische Reaktionen auf Nahrungsmittel können sich auf unterschiedliche Weise äußern: Hautausschlag, Nesselsucht, Bauchschmerzen, Erbrechen, Durchfall, Atembeschwerden oder Kreislaufprobleme. Insbesondere Erdnuss und Schalenfrüchte können zu schweren allergischen Reaktionen führen.

### Was bedeutet Toleranzentwicklung?

Viele Kinder mit einer Nahrungsmittelallergie verlieren Ihre Allergie über die Zeit (Toleranzentwicklung), während andere sie bis ins Jugendlichen- oder Erwachsenenalter behalten. Die meisten Kinder mit einer Hühnerei- oder Kuhmilchallergie können das Nahrungsmittel nach 1-2 Jahren wieder problemlos essen. Dagegen verlieren nur ca. 10-20% der Kinder Ihre Erdnuss- oder Schalenfruchtallergie. Wir verstehen bis heute nicht, warum es diesen Unterschied zwischen den verschiedenen Allergenen gibt. Wir vermuten aber, dass ein Grund für die unterschiedliche Toleranzentwicklung der unbewusste, regelmäßige Verzehr sehr geringer Mengen Kuhmilch und Hühnerei in der alltäglichen Ernährung sein könnte, da diese Allergene als Grundnahrungsmittel sehr häufig in Lebensmitteln verarbeitet werden. Für Erdnuss und Schalenfrüchte scheint dies unwahrscheinlich.

### Derzeitige Ernährungsempfehlungen

**Patient*innen mit einer Allergie** gegen Erdnuss oder Schalenfrüchte wird empfohlen, das Allergen strikt in Ihrer Ernährung zu meiden. Diese Empfehlung gilt für alle Patient*innen, unabhängig davon bei welcher Menge des Allergens es zu der allergischen Reaktion kam (individueller Schwellenwert), also z.B. bereits bei einem kleinen Krümel Nuss oder erst bei zehn Nüssen. Diese strenge Eliminationsdiät schränkt die Patient*innen im Alltag sehr ein. Wir vermuten, dass diese strenge Eliminationsdiät, wie sie derzeit für alle Patient*innen empfohlen wird, der natürlichen Toleranzentwicklung möglicherweise entgegenwirkt.

Dagegen sollen **tolerante Personen**, die keine Symptome nach dem Verzehr von Erdnuss oder Schalenfrüchten zeigen, das Nahrungsmittel regelmäßig verzehren, um das Wiederauftreten der Allergie zu verhindern.

### Zweck der Studie

Ziel dieser Studie ist es zu untersuchen

I welchen Einfluss eine strenge Eliminationsdiät im Vergleich zu einer gelockerten Eliminationsdiät (also ein regelmäßiger Verzehr geringer Allergenmengen, unterhalb des individuellen Schwellenwerts) auf die natürliche Toleranzentwicklung **bei Patient*innen mit einer Erdnuss- und/oder Schalenfruchtallergie** hat.

II Parallel möchten wir beobachten, in welchem Maße der reguläre Allergenverzehr **bei toleranten Personen** das Wiederauftreten der Allergie verhindern kann.

Außerdem werden wir untersuchen, welche Mechanismen im Körper bei der Toleranzentwicklung eine Rolle spielen. Die Ergebnisse dieses Projekts können sehr hilfreich sein, um in Zukunft neue Ernährungsempfehlungen für Patient*innen mit einer Nahrungsmittelallergie aussprechen und diese somit behandeln zu können. Insgesamt sollen (I) 120 Kinder und Erwachsene mit einer Erdnuss- und/oder Schalenfruchtallergie und (II) 120 mit einer Toleranz auf Erdnuss und/oder Schalenfrüchte in die Studie eingeschlossen werden.

Die Studie ist Teil einer interdisziplinären klinischen Forschungsgruppe „Food Allergy and Tolerance - FOOD@“. Dabei handelt sich um einen Zusammenschluss führender Institutionen der Charité - Universitätsmedizin Berlin, um die zugrundeliegenden Mechanismen bei der Entstehung und der Toleranzentwicklung von Nahrungsmittelallergien bei Kindern und Erwachsenen zu untersuchen.

Kinder ab 12 Monaten können an der Studie teilnehmen, wenn:

- der Verdacht auf eine Erdnuss- und/oder Schalenfruchtallergie besteht und daher eine orale Nahrungsmittelprovokation in unserer Klinik geplant ist.
- Ihr Kind eine bekannt Erdnuss- und/oder Schalenfruchtallergie hat und daher eine stationäre Re-Provokation durchgeführt werden soll, um zu untersuchen, ob eine Toleranzentwicklung stattgefunden hat.

Falls sich bei der Provokation herausstellt, dass eine Toleranz vorliegt, kann Ihr Kind teilnehmen. Stellt sich bei der Provokation heraus, dass eine Allergie besteht, kann Ihr Kind ebenfalls teilnehmen, sofern Symptome erst ab einer bestimmten Gabe der Provokation auftraten (ab etwa einer Nuss).

Die Teilnahme Ihres Kindes ist freiwillig**.** Nur wenn Sie zustimmen, können Sie in die Studie einbezogen werden. Wenn Sie nicht teilnehmen möchten, beeinträchtigt dies nicht die weitere medizinische Behandlung Ihres Kindes. Sie haben keine Nachteile zu befürchten. Durch Ihre Teilnahme an der Studie entstehen Ihnen keine Kosten.

## Was genau passiert in der Studie?

Je nachdem ob im Rahmen der routinemäßigen Nahrungsmittelprovokation Ihr Kind durch das ärztliche Fachpersonal als allergisch oder tolerant beurteilt wird und alle sonstigen Ein- und Ausschlusskriterien erfüllt sind, wird Ihr Kind in eine der beiden Studienarme eingeschlossen (Abbildung 1). Die Studie begleitet Sie und Ihr Kind über 1 Jahr.

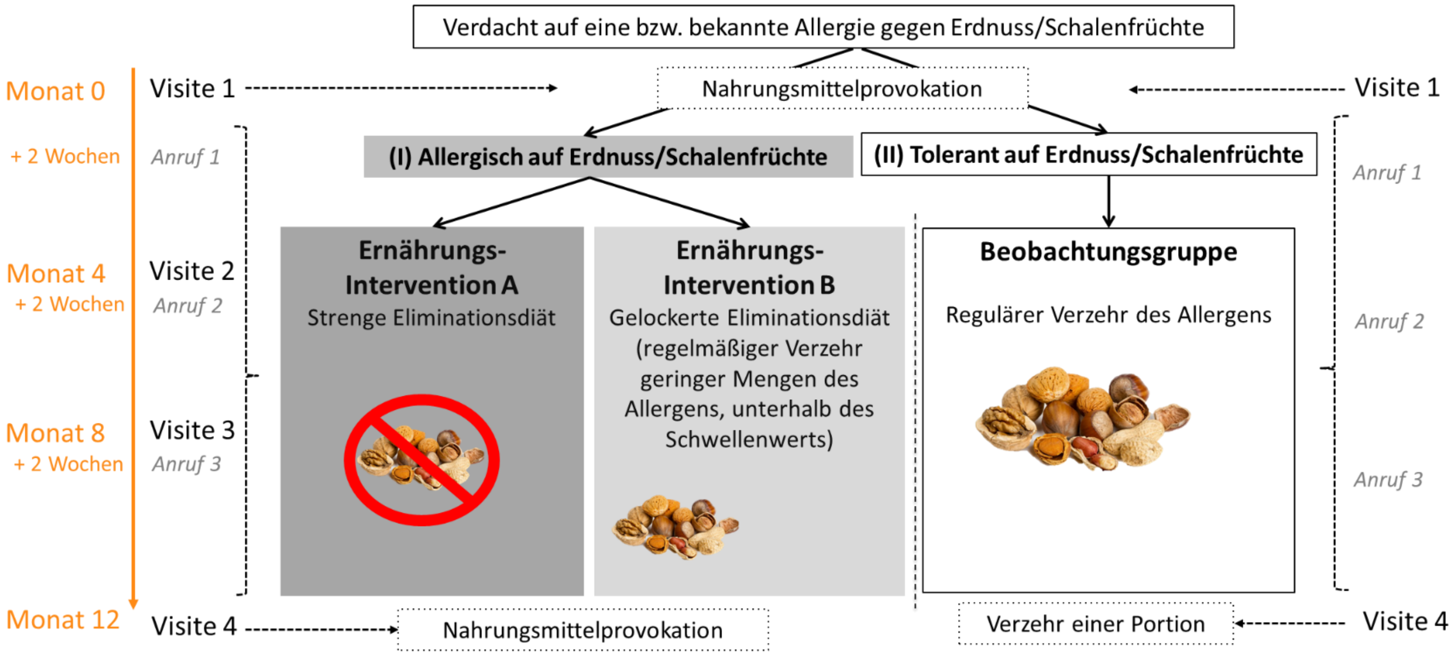


**Abbildung 1: Studiendiagramm**

## Wer kann teilnehmen?

**I Allergisch auf Erdnuss und/oder Schalenfrüchte**
**→ Ernährungs-Interventionsgruppen**

Per Zufallsprinzip wird Ihr Kind entweder der Gruppe A zugeordnet, die Erdnuss und/oder Schalenfrüchte streng meidet, oder der Gruppe B, die kleine Mengen Erdnuss und/oder Schalenfrüchte regelmäßig verzehrt (siehe Abbildung 1).

In der Gruppe A werden Sie von unseren Ernährungsfachkräften bezüglich einer standardmäßigen, strengen Eliminationsdiät, die auch das Meiden von Produkten mit „Spurenhinweisen“ auf das entsprechende Allergen beinhaltet, beraten.

In der Gruppe B werden Sie bezüglich einer gelockerte Eliminationsdiät beraten. Dabei sollen geringe Mengen an Erdnuss und/oder Schalenfrüchte unterhalb des individuellen Schwellenwerts mindestens 3-mal pro Woche, vorzugsweise täglich, verzehrt werden. Begonnen wird dabei weit unterhalb des individuellen Schwellenwerts (1/100). Nach 4 und 8 Monaten, wird die erlaubte Verzehrsmenge jeweils erhöht. Sie erhalten von uns genaue Informationen welche Produkte in welcher Menge verzehrt werden dürfen. Sobald Ihr Kind eine bestimmte Menge des Allergens verzehrt, dürfen auch Produkte mit „Spurenhinweis“ auf das entsprechende Allergen verzehrt werden.

Nach der Eingangsuntersuchung (V1), die im Rahmen der Nahrungsmittelprovokation durchgeführt wird, werden Sie und Ihr Kind im Verlauf der Studie noch zu weiteren drei Besuchen an unser Studienzentrum kommen (V2, V3 und V4). Bei der Abschlussvisite (V4) erfolgt eine erneute Nahrungsmittelprovokation, um den Erfolg der Ernährungsintervention zu untersuchen, also ob sich an der Reaktion Ihres Kindes auf Erdnuss und/oder Schalenfrüchte etwas geändert oder sich evtl. eine Toleranz entwickelt hat.

**II Tolerant auf Erdnuss und/oder Schalenfrüchte**
**→ Beobachtungsgruppe**

Sie erhalten von unseren Ernährungsfachkräften eine Beratung, bezüglich einer standardmäßigen Einführung von Erdnuss und/oder Schalenfrüchten in die Ernährung Ihres Kindes. Dies beinhaltet die Empfehlung Erdnuss und/oder Schalenfrüchte bzw. Produkte, die diese Nahrungsmittel enthalten, mindestens 3-mal pro Woche zu verzehren.

Nach der Eingangsuntersuchung (V1), die im Rahmen der Nahrungsmittelprovokation durchgeführt wird, werden Sie und Ihr Kind im Verlauf der Studie noch zu einem weiteren Besuch an unser Studienzentrum kommen (V4). Bei der Abschlussvisite (V4) wird Ihr Kind gebeten, die Menge des Nahrungsmittels unter ärztlicher Aufsicht zu verzehren, die es bereits bei der Nahrungsmittelprovokation zu Studieneinschluss vertragen hatte, um zu überprüfen, ob die Toleranz nach wie vor besteht.

**Studienablauf**

Alle Maßnahmen und Untersuchungen, die in der Studie durchgeführt werden, werden in den folgenden Abschnitten erklärt. Im Verlauf der Studie werden Sie dreimal telefonisch kontaktiert, um die Ernährung Ihres Kindes und die Verträglichkeit zu erfassen (PC1, PC2, PC3). Hierfür werden Sie zusätzlich gebeten fortlaufend ein Tagebuch zu führen (nur Interventionsgruppen), sowie zu vier Zeitpunkten Fragebögen auszufüllen.

Zu Beginn, während und am Ende der Studie bitten wir Sie einen Fragebogen zur Lebensqualität und dem Ernährungsverhalten auszufüllen.

Anamnese und körperliche Untersuchung

Bei jeder Visite (V1-V4) führen wir eine Anamnese (Erhebung der Krankengeschichte) durch und werden Ihr Kind körperlich untersuchen, vergleichbar mit einer Routineuntersuchung beim kinderärztlichen Fachpersonal. Besonderes Augenmerk werden wir dabei auf das Vorliegen und den Verlauf allergischer Erkrankungen und den Hautstatus Ihres Kindes in Bezug auf eine atopische Dermatitis legen. Hierfür werden wir den Wasserverlust der Haut (transepidermaler Wasserverlust, TEWL) messen. Dieser kann als Maß für die Einschränkung der Hautbarrierefunktion herangezogen werden und wird auf der Hautoberfläche bestimmt. Es handelt sich dabei um eine nur wenige Sekunden andauernde, schmerz- und belastungsfreie Untersuchung, die wir bei jeder Visite durchführen werden.
Die Untersuchungen werden von kinderallergologisch geschulten Ärzt*innen durchgeführt. Selbstverständlich besteht genügend Zeit um Ihre individuellen Fragen zu beantworten. Darüber hinaus werden wir Sie bitten einen Fragebogen zum Gesundheitszustand Ihres Kindes auszufüllen.

Hautpricktest

Bei allen Teilnehmer*innen wird ein Hautpricktest durchgeführt. Mit diesem Hauttest können wir Ihren Sensibilisierungsstatus (Allergiebereitschaft z.B. gegenüber Nahrungsmitteln oder Pollen) bestimmen. Der Hautpricktest zählt zu den üblichen Untersuchungsmethoden in der medizinischen Praxis bei dem Verdacht auf Nahrungsmittelallergien oder zur Verlaufskontrolle bei einer bestehenden Nahrungsmittelallergie. Dabei werden verschiedene Nahrungsmittelallergene (z.Bsp Erdnuss) und ggf. inhalative Allergenen mit einer kleinen Nadel unter die Haut am Unterarm oder dem Rücken gebracht (praktisch schmerzlos).
Nach 15 Minuten wird der Test abgelesen, wobei eine lokale Rötung und Quaddelbildung ein positives Testergebnis liefert.

Gewinnung von Blutproben

Die Blutentnahme gehört zu den üblichen Untersuchungsmethoden in der medizinischen Praxis bei dem Verdacht auf Nahrungsmittelallergien oder zur Verlaufskontrolle bei einer bestehenden Nahrungsmittelallergie. Ihrem Kind wird zu Beginn (V1) und am Ende (V4) der Studie ca. 5-15 ml Blut (etwa ½ - 1,5 Esslöffel, je nach Alter Ihres Kindes) entnommen. Erfolgt im Rahmen der Visite eine Nahrungsmittelprovokation, können wir das Blut über den Zugang bei Ihrem Kind entnehmen, der dabei routinemäßig gelegt wird, um im Notfall sofort Medikamente geben zu können. Die Blutentnahme im Rahmen der Nahrungsmittelprovokation (V1, V4) erfolgt zu Beginn beim Legen des routinemäßigen, venösen Zugangs sowie nach dem Auftreten einer allergischen Reaktion aus dem bereits liegendem Zugang.

Das Blut wird verwendet, um wie in der Routinediagnostik festzustellen, ob verschiedene Allergieantikörper gegen Erdnuss und/oder Schalenfrüchte und ggf. weitere Nahrungsmittelallergene vorliegen. Das restliche Blut wird für weitere verschiedene Labortests verwendet. Hierbei sollen Zellen, Antikörper und Botenstoffe, sowie erblich bedingte (genetische) Faktoren untersucht werden, die mit der Entwicklung der Toleranz einhergehen und solche, die die Schwere einer allergischen Reaktion voraussagen können. Genetische Faktoren und Umweltfaktoren bestimmen gemeinsam das Risiko, eine allergische Erkrankung zu entwickeln, aber vermutlich auch auf die natürliche Toleranzentwicklung. Es ist wichtig herauszufinden, welche Gene daran an der Toleranzentwicklung beteiligt sind und welche Auswirkungen sie haben. Erbvarianten, die krankheitsrelevant sind, kommen bei Patient*innen häufiger vor, als bei Gesunden. Bei einer das ganze Erbgut umfassenden Untersuchung, der genomweiten Sequenzierung, können wir alle erblichen Varianten im Erbgut (Genom) identifizieren und prüfen, ob sie bei den Betroffenen besonders häufig vorkommen und welche Auswirkungen sie haben. Dabei werden die Daten von Hunderten bis Tausenden Patient*innen und Gesunden verglichen, sodass die Ergebnisse nur für die Gesamtgruppe und nicht für einzelne Patient*innen ausgewertet werden. Da wir in vorhergehenden Studien herausgefunden haben, dass die Zellen des Abwehrsystems (Immunzellen) im Blut eine besonders wichtige Rolle bei der Allergieentstehung spielen, werden wir außerdem in derselben Blutprobe diese Immunzellen untersuchen. Ihre Funktion hängt davon ab, welche Gene aktiv sind. Das können wir mit neuesten Technologien, wie der sog. Einzelzell-Sequenzierung, feststellen. Darüber hinaus wollen wir genom-weit „molekulare Schalter“ (z.B. DNS-Methylierung) untersuchen, mit denen Gene aktiviert oder deaktiviert werden (Epigenetik). Durch diese Untersuchungen wollen wir herausfinden, welche erblichen Veränderungen zur Allergie- und Toleranzentstehung beitragen und wie sie die Zusammensetzung und Funktion der Immunzellen beeinflussen.

Alle Ergebnisse, die in der Routinediagnostik von Bedeutung sind, werden Ihnen selbstverständlich mitgeteilt und mit Ihnen besprochen. Ziel ist es eine diagnostische Möglichkeit zu finden, um die Nahrungsmittelprovokation möglicherweise zukünftig umgehen zu können.

Gewinnung von Hautabstrichen, Stuhl-, Speichel- und Hausstaubproben

Bei jeder Visite (V1-V4) werden Speichel-, Stuhl- und Hausstaubproben eingesammelt. Bei der Eingangs- und Abschlussvisite (V1 und V4) werden außerdem Hautabstriche durchgeführt. Die Hautabstriche und Speichelproben werden im Laufe der Visite gewonnen, die Stuhl- und Staubproben können von Ihnen zu Hause gewonnen und zur jeweiligen Visite mitgebracht werden.

Die Haut und der Darm sind mit vielen verschiedenen Bakterien besiedelt, die uns Menschen nicht schaden. In ihrer Gesamtheit nennen wir sie „Haut-“ oder „Darmflora“. Die Bakterienflora kann die Immunantwort des Menschen und speziell die Entwicklung allergischer Erkrankungen beeinflussen. Mit Hilfe der Stuhlproben und Hautabstriche, möchten wir die hautspezifische und darmspezifische Bakterienflora und deren Einfluss auf die Toleranzentwicklung bei Nahrungsmittelallergien untersuchen. Hierfür wird ein Hautabstrich an zwei verschiedenen Stellen der Haut mit einem feuchten Wattebausch bei Ihrem Kind durchgeführt.

Der Stuhl wird abhängig vom Alter Ihres Kindes mit bereitgestellten Probenbehältern aus der Windel, dem Töpfchen oder einem Auffangpapier gewonnen. Außerdem soll der Einfluss der Ernährung und weiterer Umweltfaktoren insbesondere der Besitz eines Hundes hieraus untersucht werden.

Wir wissen, dass Allergene im Hausstaub eine wichtige Rolle bei Nahrungsmittelallergien spielen. Daher bitten wir sie Hausstaubproben an bestimmten Orten Ihres Wohnraums mit einem speziellen Staubsaugeraufsatz zu sammeln.

Die Untersuchung von Speichel findet in verschiedenen Bereichen der Medizin bereits Anwendung. Wir wollen untersuchen, ob Speichel auch für die Allergiediagnostik geeignet ist und insbesondere eine Toleranzentwicklung voraussagen kann. Dies ist insbesondere interessant, da die Speichelgewinnung unkompliziert und schmerzfrei durchgeführt werden kann.

Hierfür werden wir mittels eines langen Watteträgers, an dem ihr Kind einfach lutschen kann, Speichel aus der Mundhöhle gewinnen.

Orale Nahrungsmittelprovokation
Auch die orale Nahrungsmittelprovokation ist ein Standardverfahren in der Routinediagnostik, um eine Verdachtsdiagnose auf Nahrungsmittelallergie zu bestätigen oder um bei bestätigter Nahrungsmittelallergie zu untersuchen, ob Ihr Kind im Laufe der Zeit tolerant geworden ist und das Nahrungsmittel möglicherweise wieder verträgt. Routinemäßig werden orale Nahrungsmittelprovokationen stationär durchgeführt.
Die Provokation wird in der Regel Placebo-kontrolliert und doppelblind durchgeführt. Dabei werden Ihrem Kind aufsteigende Mengen des Nahrungsmittels, das im Verdacht steht, die Allergie auszulösen, verabreicht. Das verdächtige Nahrungsmittel wird dabei in ein verträgliches Lebensmittel (z.B. Pudding) untergemischt. Zum Vergleich gibt es aber auch Portionen, die das verdächtige Nahrungsmittel nicht enthalten (sogenanntes Placebo). Doppelblind bedeutete, dass weder Sie noch das anwesende ärztliche Fachpersonal beim Test wissen darf, in welcher Mahlzeit das „verdächtige" Nahrungsmittel enthalten ist. Die Durchführung pro Nahrungsmittel dauert zwei Tage, während und nach jeder Mahlzeit wird Ihr Kind sorgfältig überwacht, um zu sehen, ob allergieverdächtige Reaktionen auftreten. Der Nahrungsmittelprovokationstest wird gestoppt, wenn eine klinische Reaktion auf das provozierte Nahrungsmittel auftritt. Routinemäßig wird vor Beginn der Nahrungsmittelprovokation ein Zugang gelegt, um im Notfall sofort Medikamente geben zu können.

## Was geschieht mit den Bioproben die wir gewonnen haben?

Die im Rahmen der Studie entnommenen Bioproben werden pseudonymisiert (verschlüsselt) und in dieser Form an folgende Labore zu den oben genannten Untersuchungszwecken übermittelt. Den Forschern ist kein Rückschluss auf Ihr Kind möglich. Die Bioproben werden nicht in den Laboren gelagert, sondern nach Analyse vernichtet:

- Pädiatrie m. S. Pneumologie und Immunologie mit Intensivmedizin, Charité - Universitätsmedizin Berlin
  Augustenburgerplatz 1, 13353 Berlin
- Klinik für Dermatologie, Venerologie und Allergologie; Charité - Universitätsmedizin Berlin
  Charitéplatz 1, 10117 Berlin
- Institut für Mikrobiologie und Infektions-Immunologie; Charité - Universitätsmedizin Berlin
  Hindenburgdamm 30, 12203 Berlin
- Max-Delbrück-Centrum, Charité - Universitätsmedizin Berlin; Robert-Rössle-Str. 10, 13092 Berlin
- Experimental and Clinical Research Center des Max-Delbrück-Centrum und der Charité
  Lindenberger Weg 80, 13125 Berlin
- Berlin-Brandenburg Center für Regenerative Therapien (BCRT); Augustenburger Platz 1, 13353 Berlin
- Thermo Fisher Scientific, Servicelabor,
  Munzinger Straße 7, 79111 Freiburg
- Labor Berlin, Sylter Str. 2, 13353 Berlin

Ein Teil der Bioproben wird über einen Zeitraum von 10 Jahren nach Abschluss der Studie und Veröffentlichung der Ergebnisse ebenfalls pseudonymisiert im Labor der Pädiatrie m. S. Pneumologie und Immunologie mit Intensivmedizin (Augustenburgerplatz 1, 13353 Berlin) und im Labor der Klinik für Dermatologie, Venerologie und Allergologie (Charitéplatz 1, 10117 Berlin) gelagert, für die (unter anderem auch genetische) Analysen, in weiteren Projekten, die aus neuen wissenschaftlichen Erkenntnissen der nächsten Jahre hervorgehen.
Zugang zu den Bioproben hat nur autorisiertes Studienpersonal.
Bei jeder Erhebung, Speicherung und Übermittlung von Daten aus Biomaterialien im Rahmen von Forschungsprojekten bestehen Vertraulichkeitsrisiken (z. B. die Möglichkeit, Sie zu identifizieren), insbesondere im Hinblick auf die Information zur Erbsubstanz. Diese Risiken lassen sich nicht völlig ausschließen und steigen, je mehr Daten miteinander verknüpft werden können. Die Studienleitung versichert Ihnen, alles nach dem Stand der Technik Mögliche zum Schutz der Privatsphäre zu tun und Bioproben nur an Projekte weiterzugeben, die ein geeignetes Datenschutzkonzept vorweisen können.

## Was geschieht mit den Daten, die wir erhoben haben?

Die Speicherung und Analyse der Daten (inklusive der (epi)genetischen Daten) erfolgt in Zusammenarbeit mit dem:

- Institut für Sozialmedizin, Epidemiologie und Gesundheitsökonomie, Charité - Universitätsmedizin Berlin
  Charitéplatz 1, 10117 Berlin
- Klinik für Dermatologie, Venerologie und Allergologie, Charité - Universitätsmedizin Berlin
  Charitéplatz 1, 10117 Berlin
- Institut für Physiologie, Charité - Universitätsmedizin Berlin; Charité Platz 1, 10117 Berlin
- Experimental and Clinical Research Center des Max-Delbrück-Centrum und der Charité
  Lindenberger Weg 80, 13125 Berlin

Alle Studiendaten werden nur pseudonymisiert (d.h. ein Rückschluss auf ihr Kind ist dort nicht möglich) weitergegeben. Personenbezogene Daten verbleiben ausschließlich im Studienzentrum der Klinik für Pädiatrie m. S. Pneumologie und Immunologie mit Intensivmedizin der Charité. Studienrelevante Unterlagen werden 10 Jahre nach Abschluss der Studie und Veröffentlichung der Ergebnisse im Studienzentrum aufbewahrt und anschließend auf sichere Weise entsorgt. Die gesammelten Daten werden elektronisch archiviert. Zugang zu den Daten hat nur autorisiertes Studienpersonal.

## Was sind die Vorteile einer Teilnahme?

Die Studie wird von allergologisch erfahrenen Ärzt*innen und Ernährungsfachkräften durchgeführt, die Sie und Ihr Kind intensiv betreuen. Die klinisch indizierte Allergiediagnostik (Messung von Allergieantikörpern, Nahrungsmittelprovokation) hilft bei der Beurteilung bestehender oder neu hinzugekommener Allergien. Sie kann im Falle einer Erdnuss- und/oder Schalenfruchtallergie aber auch helfen zu beurteilen, ob ihr Kind das entsprechende Nahrungsmittel wieder verträgt.
Falls Ihr Kind eine bestehende Erdnuss- und/oder Schalenfruchtallergie hat, besteht die Möglichkeit, dass es mit der neuen Ernährungsempfehlung schneller eine Toleranz gegen Erdnuss bzw. Schalenfrüchte entwickelt.

## Was ist der Nutzen der Teilnahme und der Studie für die Allgemeinheit?

Die weitergehenden Untersuchungen, wie die Analyse des Mikrobioms des Darms und der Haut sowie die Immunantwort des Körpers auf Nahrungsmittelallergene, werden das Verständnis der Entstehung und den Verlauf von allergischen Erkrankungen und der Toleranzentwicklung verbessern. Sie können längerfristig zur Entwicklung verbesserter Möglichkeiten der Früherkennung und Behandlung von Nahrungsmittelallergien beitragen. Die aktuellen Ernährungsempfehlungen für Patient*innen könnten ggf. aktualisiert werden und Patient*innen mit einer persistenten Nahrungsmittelallergie müssten das Nahrungsmittel nicht mehr streng meiden, was die Lebensmittelauswahl erleichtern und zu einer verbesserten Lebensqualität führen würde.

## Was sind mögliche Risiken einer Teilnahme?

Ihr Kind könnte sich bei der Blutabnahme leicht unwohl fühlen. Zur Betäubung der Haut kann auf Wunsch eine anästhesierende Creme aufgetragen werden. An der Stelle der Blutentnahme kann ein leichter Bluterguss entstehen. Infektionen sind unter Standardhygienebedingungen extrem selten.
Bei dem Hautpricktest tritt häufig lokaler Juckreiz auf, allergische Allgemeinreaktionen sind extrem selten. Der Hautpricktest wird nur von allergologisch geschultem Personal und unter Überwachung durchgeführt.
Bei der Teilnahme Ihres Kindes an einem Nahrungsmittelprovokationstest kann es unter anderem zu folgenden allergischen Reaktionen kommen: Juckreiz, Quaddeln (Nesselsucht), Erbrechen, Durchfall, Husten, pfeifende Atmung oder Verschlechterung einer bestehenden atopischen Dermatitis („Neurodermitis“). Schwere Reaktionen wie Anaphylaxie (Atemnot oder schockartige allergische Reaktion) treten nur in seltenen Fällen auf. Alle Nahrungsmittelprovokationstests werden von geschultem ärztlichen Fachpersonal streng überwacht, sodass allergische Reaktionen sofort behandelt werden können und eine Notfallversorgung ist zu jedem Zeitpunkt sichergestellt. Routinemäßig wird daher vor Beginn der Nahrungsmittelprovokation ein Zugang gelegt, um im Notfall sofort Medikamente geben zu können.

Bei der gelockerten Eliminationsdiät erwarten wir keine allergischen Symptome, da während der Provokation gezeigt werden konnte, dass Ihr Kind diese und weitaus höhere Mengen des Allergens verträgt. Zur Sicherheit wird die erste vertragene, sowie die zwei gesteigerten Verzehrsmengen des Allergens unter ärztlicher Aufsicht gegeben. Zwar können wir allergische Reaktionen nicht zu 100% ausschließen, schwere allergische Reaktionen sind jedoch sehr unwahrscheinlich.

## Was sind Umstände, die zum Abbruch der Studienteilnahme führen können?

Ihre Teilnahme an der Studie ist freiwillig und nur mit Ihrer schriftlichen Zustimmung auf der Einwilligungserklärung möglich. Sie haben jederzeit das Recht ohne Nennung von Gründen, die Teilnahme Ihres Kindes an der Studie zu beenden. Auch das studienärztliche Fachpersonal hat das Recht, die Studienteilnahme Ihres Kindes aus Sicherheitsgründen, wegen Änderung der Maßnahmen und aus anderen medizinischen Gründen zu beenden. Ein Abbruch beeinträchtigt in keiner Weise die weitere medizinische Behandlung und Betreuung Ihres Kindes. Sie haben keine Nachteile zu befürchten.

## Wie werden meine Daten geschützt?

Im Rahmen der Studie werden personenbezogene Daten erhoben, verarbeitet und ggf. pseudonymisiert weitergegeben. Außerdem werden im Rahmen der Studie Bioproben (Blut, Speichel, Haut, Stuhl) erhoben und verarbeitet (siehe Abschnitt „Was geschieht mit den Bioproben die wir gewonnen haben?“). Die

Verarbeitung der Daten erfolgt gemäß den einschlägigen rechtlichen Bestimmungen insbesondere der Datenschutzgrundverordnung (DSGVO), dem Berliner Datenschutzgesetz (BerlDS) sowie §25 des Berliner Gesetz zur Regelung des Krankenhausrechts (LKG). Durch Ihre Unterschrift auf der Einwilligungserklärung erklären Sie sich damit einverstanden, dass die Studienleitung und deren Mitarbeiter die personenbezogenen Daten Ihres Kindes zum Zweck der o.g. Studie erheben und verarbeiten dürfen. Personenbezogene Daten sind z.B. der Name Ihres Kindes, Geburtsdatum, Ihre Adresse und Daten zur Gesundheit oder Erkrankung oder andere persönliche Daten Ihres Kindes, die während Ihrer Teilnahme an der Studie oder bei einer der Folgeuntersuchungen zweckgebunden erhoben wurden. Die Studienleitung wird die personenbezogenen Daten Ihres Kindes ausschließlich für Zwecke der Verwaltung und Durchführung der Studie verwenden und diese, einem Pseudonym zugeordnet, für Zwecke der Forschung und statistischen Auswertung verwenden. Auf den Codeschlüssel, der es erlaubt, die pseudonymisierten Daten mit Ihrem Kind in Verbindung zu bringen, haben nur die Studienleitung und deren Mitarbeiter Zugriff. Dritte wie z.B. Sponsoren und deren befugte Mitarbeiter erhalten nur Einblick in personenbezogene Unterlagen, um die Qualität der Durchführung der Studie abzusichern. Bitte beachten Sie, dass die Ergebnisse der Studie in der medizinischen Fachliteratur veröffentlicht werden können, wobei die Identität Ihres Kindes jedoch anonym bleibt, so dass die Veröffentlichung ohne personenbezogene Daten erfolgt. Für den Schritt der Anonymisierung erfragen wir Ihre Einwilligung, da es sich um eine Verarbeitung im Sinne von Art. 4 Absatz 2 DSGVO handelt. Da nach einer Anonymisierung ein Personenbezug nicht mehr herstellbar ist, ist der Anspruch auf die im folgenden aufgeführten Rechten (Auskunft, Berichtigung oder Löschung) nicht durchführbar.

Sie haben im Zusammenhang mit der Datenverarbeitung folgende Rechte:

Sie können jederzeit ihre erteilte Einwilligung widerrufen. Beachten Sie bitte, dass die bis dahin erfolgte Verarbeitung Ihrer Daten nicht berührt wird, Art. 7 Absatz 3 DSGVO.

Daneben haben Sie jeweils das Recht:

- auf Auskunft über alle Verarbeitungen der zur Person verarbeiteten & gespeicherten Daten und Empfänger, an die Daten weitergegeben werden oder wurden. Die Bereitstellung der Daten kann in einem maschinenlesbaren Format verlangt werden, Art.15, 20 DSGVO, auf Berichtigung unrichtiger personenbezogener Daten, Art.16 DSGVO.
- einer Weiterverarbeitung der personenbezogenen Daten Ihres Kindes zu widersprechen, die ohne Ihre Einwilligung aufgrund eines öffentlichen Interesses oder zur Wahrung berechtigter Interessen des Verantwortlichen erfolgt ist. Der Widerspruch einer Weiterverarbeitung ist zu begründen, so dass deutlich wird, dass besondere in Ihrer Person begründete Umstände das vorgenannte Interesse an einer Weiterverarbeitung überwiegt, Art. 21 DSGVO.
- auf Löschung/Vernichtung unter der Voraussetzung, dass bestimmte Gründe vorliegen. Dies ist insbesondere der Fall bei unrechtmäßiger Verarbeitung, wenn die Daten/Bioproben zu dem Zweck zu dem sie erhoben oder verarbeitet wurden nicht mehr notwendig sind, Sie die Einwilligung widerrufen und eine anderweitige Rechtsgrundlage für die Datenverarbeitung nicht gegeben ist oder anstelle des vorbenannten Widerspruchs nach Art. 21 DSGVO unter den dort genannten Voraussetzungen. Sofern die Löschung/Vernichtung die Ziele eines im wissenschaftlichen Interesse durchgeführten Forschungsprojektes zu Nichte machen oder wesentlich erschweren würde, besteht kein Recht auf Löschen/Vernichtung, Art. 17 Absatz III. Nach Ablauf einer Aufbewahrungszeit von 10 Jahren nach Beendigung der Studie und Veröffentlichung der Ergebnisse werden die personenbezogenen Daten/Bioproben Ihres Kindes gelöscht/vernichtet.
- auf Einschränkung der Verarbeitung personenbezogener Daten, insbesondere wenn die Verarbeitung unrechtmäßig ist & Sie die Einschränkung anstelle des Löschens verlangen (siehe dort) oder solange streitig ist, ob die Verarbeitung personenbezogener Daten rechtmäßig erfolgt, Art.18 DSGVO.

Zur Wahrnehmung der vorgenannten Rechte wenden Sie sich bitte an die für die Verarbeitung der personenbezogenen Daten verantwortliche Stelle: Charité Universitätsmedizin (Charitéplatz 1, 10117 Berlin) vertreten durch die Studienleitung:

Prof. Dr. med. Kirsten Beyer
Dr. rer nat. Valérie Trendelenburg
Kinderallerologisches Studienzentrum
Charité – Universitätsmedizin Berlin
Augustenburger Platz 1, 13353 Berlin
Tel.: 030 450 566037

Bei Anliegen zur Datenverarbeitung und zur Einhaltung der datenschutzrechtlichen Anforderungen können Sie sich auch an folgende Stelle wenden:
Stabsstelle Datenschutz
Charitéplatz 1, 10117 Berlin
Tel.: 030 450 580016 | E-Mail: datenschutz@charite.de

Für den Fall, dass Sie eine Datenverarbeitung für rechtswidrig halten, haben Sie neben der Inan-

spruchnahme gerichtlicher Hilfe die Möglichkeit. Beschwerde einzureichen bei der für die Charité Universitätsmedizin Berlin zuständigen Aufsichtsbehörde:
Berliner Beauftragte für Datenschutz und Informationsfreiheit
Friedrichstraße 219, 10969 Berlin
Tel.: +49 30 13889-0 | Fax: +49 30 2155050
E-Mail: mailbox@datenschutz-berlin.de

Die Daten-/Bioprobenerhebung zu wissenschaftlichen Zwecken ist kosten- und zeitintensiv, sodass die Verwendung bereits erhobener Daten/Bioproben dazu beitragen kann, beispielsweise neu entwickelte Analysemethoden schneller auf ihren potenziellen medizinischen Nutzen hin zu untersuchen. Daher erfragen wir in der Einwilligungserklärung Ihre Zustimmung zur Verarbeitung der Daten/Bioproben in konkreten Anschlussstudien bzw. um Zustimmung zu einer späteren Kontaktaufnahme zu dem Zweck, Ihre Einwilligung zur Verarbeitung der bereits erhobenen Daten/Bioproben oder zur Erhebung neuer Daten/Bioproben im Rahmen einer möglichen weiteren, zum gegenwärtigen Zeitpunkt noch nicht festgelegten Studie.

### Versicherungsschutz

Für diese Studie wurde keine spezielle Versicherung für die Patient*innen abgeschlossen. Die an der Studie beteiligten Mitarbeiter der Charité (Studienärzte und -ärztinnen, Ernährungsfachkräfte, Studienschwestern und -pfleger etc.) sind durch die Betriebshaftpflichtversicherung der Charité gegen Haftpflichtansprüche, welche aus ihrem schuldhaften Verhalten resultieren könnten, versichert.

### Wer finanziert und organisiert das Forschungsprojekt?

Diese Studie wird als Teil der klinischen Forschungsgruppe „Food Allergy and Tolerance - FOOD@“ durch Drittmittel der Deutsche Forschungsgesellschaft (DFG) finanziert.

### Haben Sie noch Fragen?

Wir hoffen, Ihnen alle Fragen zur Studie zufriedenstellend beantwortet zu haben. Bei Fragen oder Bedenken hinsichtlich Ihrer Studienteilnahme, insbesondere auch zu möglichen Risiken, oder wenn Sie zu irgendeinem Zeitpunkt mit bestimmten Punkten der Studie unzufrieden sind, können Sie sich jederzeit an uns wenden.

**Ihr Team des Kinderallergologischen Studienzentrums!**

**Email:** tina-studie@charite.de
**Tel:** 030/450 516 466 (mit Anrufbeantworter)

**Prof. Dr. med. Kirsten Beyer**
Leiterin des Kinderallergologischen Studienzentrums

**Wir danken Ihnen für Ihre Zeit und würden uns freuen, wenn Sie sich für die Teilnahme an unserem Forschungsprojekt entscheiden!**

CHARITÉ

CharitéCentrum für Frauen-, Kinder- und Jugendmedizin mit Perinatalzentrum und Humangenetik

**Klinik für Pädiatrie mit Schwerpunkt Pneumologie und Immunologie mit Intensivmedizin**
Klinikdirektor: Prof. Dr. med. Marcus Mall
Sektion Kinderallergologisches Studienzentrum
Leitung: Prof. Dr. med. Kirsten Beyer
Telefon Sekretariat: 030 – 450 566 037
Telefon Studienteam: 030 – 450 516 466
Fax: 030 – 450 566 931
E-Mail Studienteam: tina-studie@charite.de
www.charite-ppi.de

Information sheet for adolescents aged 13-17 years

# Teilnehmer*inneninformation für Jugendliche im Alter von 13-17 Jahren

für die Teilnahme an der Studie

## Förderung der Toleranzentwicklung durch „Nicht-Vermeidung“ zur Verhinderung einer persistierenden Nahrungsmittelallergie

**Hallo!**

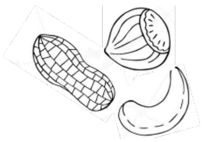

**da du eine bekannte Erdnuss- und/oder Schalenfruchtallergie („Nüsse“) hast oder ein Verdacht darauf besteht, möchten wir dich einladen an unserer wissenschaftlichen Untersuchung (Studie) teilzunehmen. Wir untersuchen, ob die Ernährung einen Einfluss darauf hat, dass deine Allergie wieder verschwindet.**

**Damit du weißt, was bei dieser Studie auf dich zukommt, bitten wir dich, dieses Informationsblatt genau durchzulesen. Deine Eltern bekommen auch ein Informationsblatt, damit du dich mit ihnen über die Studie unterhalten kannst und sie dir helfen können, dich zu entscheiden.**

### Was sind Nahrungsmittelallergien?

In vielen Ländern leiden bis zu 8% der Kinder und 5% der Erwachsenen an einer Nahrungsmittelallergie. Allergien gegen Erdnüsse und Schalenfürchte sind dabei häufig. Zu den Schalenfrüchten zählt man die Haselnüsse, Walnüsse, Mandeln, Cashewnüsse, Pekannüsse, Paranüsse, Macadamianüsse und Pistazien. Eine Allergie kann sich bei Dir auf unterschiedliche Weise zeigen, ganz typisch sind: Hautausschlag, Nesselsucht, Bauchschmerzen, Erbrechen, Durchfall, Atembeschwerden oder Kreislaufprobleme. Die Reaktionen auf Erdnuss oder Schalenfürchte verlaufen manchmal auch sehr schwer. Die schwerste Reaktion bezeichnet man als Anaphylaxie (Schock). streng meiden. Bei einer Allergie gegen Erdnuss oder Schalenfrüchte, soll man das verantwortliche Nahrungsmittel streng meiden. Diese strenge Diät kann ganz schön anstrengend sein, da Erdnuss und Schalenfrüchte häufig auf Verpackungen genannt werden.

Manche Kinder und Jugendliche mit einer Nahrungsmittelallergie verlieren diese (=Toleranz), manche behalten sie auch. Wir verstehen bis heute nicht warum.

### Was möchten wir herausfinden?

Wir möchten herausfinden, ob die Ernährung eine Rolle dabei spielt, warum manche Kinder Ihre Allergie verlieren und andere nicht und was genau dabei im Körper passiert.

### Wer kann an der Studie teilnehmen?

Du kannst an der Studie teilnehmen, weil du wegen einer Untersuchung deiner Nahrungsmittelallergie zu uns kommst oder bei uns schon einmal behandelt wurdest. Du musst nicht an unserer Studie teilnehmen, das ist völlig freiwillig. Du kannst auch jederzeit wieder aufhören, bei der Studie mitzumachen.

### Was ist eine Nahrungsmittelprovokation?

Eine Nahrungsmittelprovokation wird durchgeführt, wenn der Verdacht auf eine Nahrungsmittelallergie besteht oder, wenn man prüfen möchte, ob die Nahrungsmittelallergie vielleicht über die Zeit verschwunden ist. Bei der Nahrungsmittelprovokation werden die Nahrungsmittel, die bei Dir

eine Reaktion auslösen, mit Nahrungsmitteln vermischt, die dir nicht schaden (z.B. Pudding oder Apfelmus). Dabei fangen wir mit einer kleinen Menge an und geben dir danach größere Portionen, solange Du es verträgst. Während und nach jeder Portion beobachten wir Dich genau. Der Nahrungsmittelprovokationstest wird gestoppt, wenn Du eine Reaktion auf das provozierte Nahrungsmittel zeigst.

## Was genau passiert in der Studie?

Zuerst stellen wir mit einer Nahrungsmittelprovokation fest, ob Du eine Nahrungsmittelallergie hast. <u>Falls sich bei deiner Nahrungsmittelprovokation herausstellt, dass Du allergisch bist,</u> aber die Zeichen der Allergie sich erst ab einer bestimmten Menge (etwa einen Nusskern) gezeigt haben, möchten wir eine neue Ernährung ausprobieren. Wir möchten untersuchen, ob Du deine Allergie vielleicht wieder verlierst, wenn Du jeden Tag eine sehr kleine Menge Erdnuss oder Nuss isst. Insgesamt gibt es zwei Gruppen, in welche Gruppe Du kommst entscheidet das Los. <u>Gruppe 1</u> soll für ein Jahr nach wie vor eine strenge Diät durchführen. <u>Gruppe 2</u> darf kleine Portionen von Produkten, in denen Erdnuss oder Nüsse enthalten sind, ein Jahr lang verzehren. Die Menge von Erdnuss und Nüssen, die Du essen wirst, ist dabei aber noch niedriger als die Menge auf die Du bei der Nahrungsmittelprovokation reagiert hast (also z.B. ein kleiner Krümel Nuss, falls Du auf vier Nüsse reagiert hast). Wir helfen Dir und deinen Eltern dabei, welche Produkte Du genau auswählen darfst. Du besuchst uns dann noch zweimal am Studienzentrum. Nach einem Jahr untersuchen wir dann mit einer erneuten Nahrungsmittelprovokation, ob deine Allergie verschwunden ist oder nicht, oder ob sich etwas an deiner Reaktion geändert hat. Insgesamt gibt es also vier Besuche bei uns.

<u>Falls sich bei deiner Nahrungsmittelprovokation herausstellt, dass Du tolerant bist,</u> sollst Du Erdnuss oder Schalenfürchte im Alltag regelmäßig essen, um zu verhindern, dass die Allergie wiederkommt. Wir möchten dich dann ein Jahr lang beobachten und in der Zeit von Dir erfahren, wie gut es bei Dir klappt Erdnuss oder Schalenfrüchte im Alltag zu essen und sichergehen, dass Du nach einem Jahr das Nahrungsmittel immer noch problemlos verzehren kannst. Insgesamt gibt es also zwei Besuche bei un**s.**

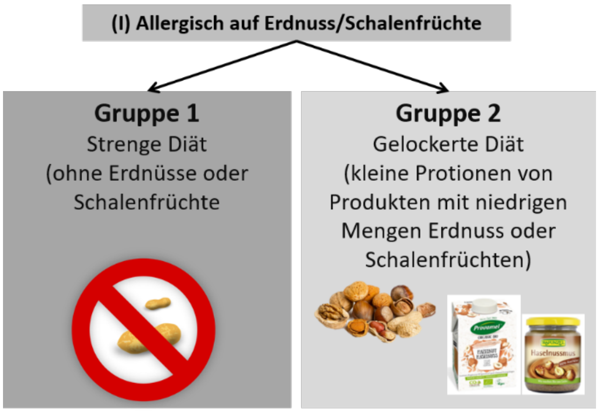


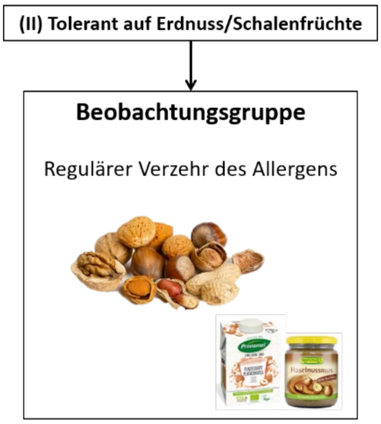


## Welche Untersuchungen machen wir noch?

Wir bitten dich, zu Beginn, während der Studie und nach einem Jahr einen Fragebogen zu Deiner Lebensqualität, sowie zur Verträglichkeit der Ernährung auszufüllen. Wir sammeln außerdem bei jedem Besuch viele Ergebnisse der Untersuchungen zu deiner Allergie und Ernährung.

Mit Hilfe verschiedener Tests möchten wir herausfinden, was durch die Ernährung mit deinem Abwehrsystem in Deinem Körper- dem Blut, deinem Speichel, der Haut und dem Darm- alles passiert. Dafür machen wir zweimal, am Anfang und Ende der Studie, eine Blutentnahme (etwa 2-3 Esslöffel) vor und nach der Nahrungsmittelprovokation, damit wir dein Blut untersuchen können. Außerdem möchten wir bei zwei Besuchen einen Abstrich mit einem Wattetupfer auf deiner Haut machen und bei jedem Besuch musst du für kurze Zeit ein Wattestäbchen in den Mund legen, damit wir deinen Speichel bekommen. Wir bitten deine Eltern zu jedem Besuch ein bisschen von deinem Stuhl und etwas von eurem Hausstaub einzusammeln.

Beim Hautpricktest werden kleine Mengen des Allergens (z.Bsp Erdnuss) auf deine Haut aufgebracht und anschließend wird die Haut leicht angekratzt. Besteht im Körper die Bereitschaft gegenüber dem getesteten Allergen zu reagieren, so führt dies in kurzer Zeit zu einer leichten Reaktion auf der Haut (Quaddeln, Rötung).

**Kann bei der Studie etwas Gefährliches passieren?**

Eine Blutentnahme oder Hauttest für einen Allergietest oder eine Nahrungsmittelprovokation gehören zu üblichen Untersuchungen, wenn der Verdacht auf eine Nahrungsmittelallergie besteht oder, wenn man prüfen möchte, ob die Nahrungsmittelallergie vielleicht über die Zeit verschwunden ist.

Wir nehmen bei der Blutentnahme noch etwas mehr Blut ab. Die Blutentnahme kann etwas weh tun. Zur Betäubung der Haut kann auf Wunsch ein Pflaster mit einer betäubenden Creme aufgetragen werden. An der Blutentnahmestelle kann ein kleiner blauer Fleck entstehen.

Beim Hauttest kann es zu leichtem Juckreiz kommen, ähnlich wie bei einem Mückenstich, der aber nach weniger Zeit wieder vergeht.

Beim Nahrungsmittelprovokationstest kannst Du z.B. Juckreiz, Erbrechen, Durchfall, Quaddeln oder eine pfeifende Atmung bekommen. Wenn Du eine Neurodermitis hast, kann sich Deine Haut verschlechtern. In sehr seltenen Fällen treten schwere Reaktionen wie eine Anaphylaxie (Schock) auf. Alle Nahrungsmittelprovokationstests werden von einem erfahrenen ärztlichen Fachpersonal streng überwacht. Die zusätzlichen Untersuchungen, wie der Abstrich mit dem Wattetupfer auf deiner Haut, das Sammeln von deinem Speichel und dem Hausstaub sind nicht gefährlich.

Während der gelockerten Diät zu Hause glauben wir nicht, dass es zu allergischen Reaktionen kommt, da Du ja Mengen isst, die noch niedriger sind als die, die Du bei der Nahrungsmittelprovokation bereits gut vertragen hast. Wir zeigen Dir und deinen Eltern natürlich, wie Du Dich am besten verhältst, falls es doch zu einer allergischen Reaktion kommt. Es ist sehr unwahrscheinlich, dass Du eine schwere allergische Reaktion bekommst.

**Wir würden uns freuen, wenn Du bei unserer Untersuchung mitmachen möchtest!**

Wenn Du mitmachst, können wir viel über Nahrungsmittelallergien lernen und allen Menschen mit Nahrungsmittelallergien besser helfen. Deine Teilnahme hilft, das Auftreten und Verschwinden von Nahrungsmittelallergien besser zu verstehen und diese in Zukunft behandeln zu können. Wir wollen erreichen, dass alle ohne Angst essen und trinken können was sie möchten, ohne dass ihr Körper allergisch reagiert.

**Wenn Du noch Fragen hast, kannst Du Dich selbstverständlich an uns wenden!**

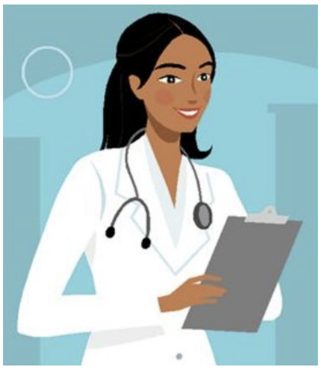

CHARITÉ

ChartéCentrum für Frauen-, Kinder- und Jugendmedizin mit Perinatalzentrum und Humangenetik

**Klinik für Pädiatrie mit Schwerpunkt Pneumologie und Immunologie mit Intensivmedizin**
Klinikdirektor: Prof. Dr. med. Marcus Mall
Sektion Kinderallergologisches Studienzentrum
Leitung: Prof. Dr. med. Kirsten Beyer
Telefon Sekretariat: 030 – 450 566 037
Telefon Studienteam: 030 – 450 516 466
Fax: 030 – 450 566 931
E-Mail Studienteam: tina-studie@charite.de
www.charite-ppi.de

Information sheet for children aged 6-12 years

# Teilnehmer*inneninformation für Kinder im Alter von 6-12 Jahren

für die Teilnahme an der Studie

## Förderung der Toleranzentwicklung durch „Nicht-Vermeidung“ zur Verhinderung einer persistierenden Nahrungsmittelallergie

### Hallo!

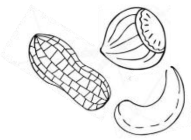

**Da du vielleicht eine Nahrungsmittelallergie gegen Erdnüsse oder eine andere Nuss hast, möchten wir dich einladen an unserer Untersuchung teilzunehmen. Wir untersuchen, ob die Ernährung eine Rolle dabei spielt, dass deine Allergie wieder verschwindet.**

**Wie alles genau funktioniert, kannst du hier nun lesen. Wenn Du noch mehr wissen möchtest, oder etwas nicht verstanden hast, dann kannst Du uns einfach fragen. Gemeinsam mit deinen Eltern kannst Du dann entscheiden, ob du bei der Untersuchung teilnehmen möchtest.**

### Was ist eine Nahrungsmittelallergie?

Manche Kinder können alles essen und trinken und werden davon nicht krank. Bei einigen Kindern funktioniert das leider nicht so gut. Sie haben eine Nahrungsmittelallergie. Wenn Sie zum Beispiel Erdnüsse essen, mag der Körper das nicht. Es kann sein, dass Sie dann Bauchweh oder Durchfall bekommen. Auch die Haut kann sich verändern und juckt vielleicht. Manchmal geht auch das Luft holen nicht mehr so gut. Wir Erwachsenen nennen diese Beschwerden eine „allergische Reaktionen.“
Manche Kinder mit einer Nahrungsmittelallergie verlieren diese wieder, manche behalten sie auch. Wir verstehen bis heute nicht warum.

### Warum machen wir die Untersuchung?

Wir möchten herausfinden, ob die Ernährung eine Rolle dabei spielt, warum manche Kinder Ihre Allergie verlieren und andere nicht und was genau dabei im Körper passiert.

### Kann ich an der Untersuchung teilnehmen?

Du kannst mitmachen, weil du wegen deiner Nahrungsmittelallergie zu uns kommst oder deswegen schon einmal bei uns warst. Du musst nicht an unserer Untersuchung teilnehmen. Du darfst entscheiden, ob du mitmachen möchtest, es ist freiwillig. Du kannst auch jederzeit wieder aufhören bei der Untersuchung mitzumachen.

### Was ist ein Esstest?

Ein Esstest wird bei Dir gemacht, weil man sich nicht sicher ist, ob Du eine Nahrungsmittelallergie hast oder ob Deine Nahrungsmittelallergie vielleicht über die Zeit verschwunden ist. Bei dem Esstest bekommst Du eine kleine Portion Pudding oder Obstbrei zu essen, in dem etwas Erdnuss oder Nuss versteckt ist. Nach einiger Zeit bekommst du etwas größere Portionen von diesem Pudding oder Obstmus. Während und nach jeder Portion gucken wir was passiert. Sobald es dir dabei nicht gut geht, hören wir natürlich auf.

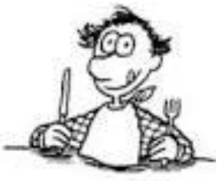

### Was genau passiert bei der Untersuchung?

Zuerst stellen wir bei dem Esstest fest, ob Du eine Nahrungsmittelallergie hast oder nicht. Wenn sich herausstellt, dass Du eine Nahrungsmittelallergie hast, aber trotzdem eine bestimmte Menge Erdnuss oder Nuss essen kannst (etwa einen halben

Nusskern), ohne dass es deinem Körper etwas ausmacht, dann möchten wir etwas ausprobieren. Wir möchten untersuchen, ob du deine Allergie vielleicht wieder verlierst, wenn Du etwa jeden Tag ganz kleine Mengen Erdnuss oder Nuss isst. Insgesamt wird es zwei Gruppen geben. In welche Gruppe Du kommst wird ausgelost. Gruppe 1 soll nach wie vor gar keine Erdnuss oder Nüsse essen und Gruppe 2 darf kleine Portionen von Lebensmitteln, in denen Erdnuss oder Nuss enthalten ist, essen. Die Menge von Erdnuss und Nuss, die Du essen wirst, ist noch niedriger als die Menge in der Portion, die Du bei dem Esstest bereits gut vertragen hat (also zum Beispiel ein kleiner Krümel Nuss). Wir helfen deinen Eltern dabei, welche Lebensmittel Du essen darfst. Du besuchst uns dann noch zweimal. Nach einem Jahr machst du nochmal einen Esstest, um zu sehen, ob deine Allergie verschwunden ist oder nicht. Insgesamt besuchst Du uns mit deinen Eltern vier Mal. Wenn sich herausstellt, dass Du keine Nahrungsmittelallergie hast, sollst Du Erdnuss oder Nuss oft essen. Damit hilft man dem Körper, dass er die Allergie nicht wiederkommt. Wir möchten dich dann ein Jahr lang beobachten und in der Zeit von Dir erfahren, wie gut es bei Dir klappt Erdnuss oder Nuss zu essen und auch sichergehen, dass Du nach einem Jahr das Nahrungsmittel immer noch problemlos essen kannst. Insgesamt besucht Du uns mit deinen Eltern zwei Mal.

## Welche Untersuchungen machen wir noch?

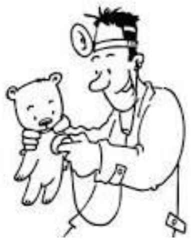

Wir bitten dich und deine Eltern zu Beginn, während der Studie und nach einem Jahr einen Fragebogen auszufüllen, da wir wissen möchten wie es dir mit oder ohne deine Allergie geht. Wir sammeln außerdem viele Ergebnisse der Untersuchungen zu deiner Allergie und Ernährung. Mit Hilfe verschiedener Tests möchten wir herausfinden, was durch die Ernährung in Deinem Körper- dem Blut, deinem Speichel, der Haut und dem Darm- alles passiert. Dafür machen wir zweimal bei einem Besuch eine Blutentnahme (etwa 2-3 Esslöffel) und untersuchen dein Blut. Außerdem möchten wir bei zwei Besuchen einen Abstrich mit einem Wattetupfer auf deiner Haut machen und du musst bei jedem Besuch für kurze Zeit ein Wattestäbchen in den Mund legen, damit wir deinen Speichel bekommen. Wir bitten deine Eltern zu jedem Besuch ein bisschen von deinem Stuhl und eurem Hausstaub mitzubringen. Beim Hautpricktest werden kleine Mengen des Allergens (z.Bsp Erdnuss) auf deine Haut aufgebracht und die Haut leicht angekratzt.

## Kann bei der Studie etwas Gefährliches passieren?

Eine Blutentnahme oder Hauttest für einen Allergietest und ein Esstest werden oft bei Kindern gemacht, bei denen nicht sicher ist, ob sie eine Nahrungsmittelallergie haben. Wir nehmen bei der Blutentnahme noch etwas mehr Blut ab. Damit du den Mückenpieks nicht merkst, können wir dir vorher ein besonderes Pflaster auf den Arm kleben. Dann tut es nicht mehr weh. Manchmal kann an der Blutentnahmestelle ein kleiner blauer Fleck entstehen. Beim Hauttest kann deine Haut leicht jucken, auch ähnlich wie bei einem Mückenpieks, der aber nach weniger Zeit wieder vergeht. Beim Esstest kannst Du Juckreiz, Erbrechen, Durchfall, Quaddeln oder eine pfeifende Atmung bekommen. Wenn Du eine Neurodermitis hast, kann sich Deine Haut verschlechtern. Nur in sehr seltenen Fällen treten schwere Reaktionen auf. Alle Esstests werden von uns begleitet und wenn es Dir nicht gut geht, kannst Du eine Medizin bekommen, damit es dir schnell wieder bessergeht. Die anderen Untersuchungen, wie der Abstrich mit dem Wattetupfer auf deiner Haut oder das Sammeln von deinem Speichel sind nicht gefährlich. Wenn du zu Hause die kleinen Portionen von Lebensmitteln isst, in denen Erdnuss oder Nuss enthalten ist, ist das für deinen Körper wahrscheinlich kein Problem, da Du ja Mengen isst, die noch niedriger sind als die, die Du bei dem Esstest bereits gut vertragen hast. Wir zeigen Dir und deinen Eltern natürlich, wie Du Dich am besten verhältst, falls es doch zu einer allergischen Reaktion kommt. Es ist sehr unwahrscheinlich, dass Du eine schwere allergische Reaktion bekommst.

## Wir würden uns freuen, wenn Du bei unserer Untersuchung mitmachst!

Wenn Du mitmachst, können wir viel über Nahrungsmittelallergien lernen und allen Menschen mit Nahrungsmittelallergien besser helfen. Deine Teilnahme hilft, das Auftreten und Verschwinden von Nahrungsmittelallergien besser zu verstehen und diese in Zukunft behandeln zu können.

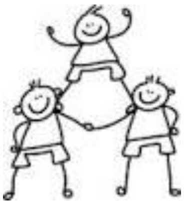

Wir wollen erreichen, dass alle ohne Angst essen und trinken können was sie möchten, ohne dass ihr Körper allergisch reagiert.

**Hast Du noch Fragen?**
