# Supplementary material for: Tolerance induction through non-avoidance to prevent persistent food allergy (TINA) in children and adults with peanut or tree nut allergy: rationale, study design and methods of a randomized controlled trial and observational cohort study
Source: Trials. 2022 Mar 28;23:236. doi: 10.1186/s13063-022-06149-4 (PMC8962184; doi:10.1186/s13063-022-06149-4)
Supplement: Supplementary file 3 — Additional file 3. Consent Form (in German). [file 13063_2022_6149_MOESM3_ESM.pdf]

CHARITÉ

ChartitéCentrum für Innere Medizin und Dermatologie

KLINIK FÜR DERMATOLOGIE,
VENEROLOGIE UND ALLERGOLOGIE
Klinikdirektor: Prof. Dr. med. K. Ghoreschi
Charitéplatz 1 | 10117 Berlin

Consent form for adults

ZERTIFIZIERTES QUALITÄTSMANAGEMENTSYSTEM ISO 9001

Klinik mit zertifiziertem Qualitätsmanagementsystem nach DIN EN ISO 9001:2008

Allergologie und Immunologie
Leiterin: Prof. Dr. med. M. Worm

Kontakt Studienteam
Tel.: +49 30 450 518 003, -305 und -417
Fax: +49 30 450 7518968
E-Mail: acc-studien@charite.de

www.derma.charite.de
www.allergie-centrum-charite.de

Einwilligungserklärung

für die Teilnahme an der Studie

Förderung der Toleranzentwicklung durch „Nicht-Vermeidung“ zur Verhinderung einer persistierenden Nahrungsmittelallergie

Hiermit erkläre ich

…………………….	…………………………….	…………………………..
Vorname	Name	Geburtsdatum

dass ich durch Herrn/Frau ………………………………………………………………………………………
(Name des Studienarztes / der Studienärztin)

mündlich und schriftlich über das Wesen, die Bedeutung und die Risiken der wissenschaftlichen Untersuchungen im Rahmen der oben genannten Studie informiert wurden und ausreichend Gelegenheit hatte, meine Fragen mit dem Studienarzt/der Studienärztin zu klären.

Mir ist bekannt, dass ich das Recht habe, meine Einwilligung jederzeit ohne Angabe von Gründen und ohne nachteilige Folgen zurückzuziehen, einer Weiterverarbeitung der Daten und Bioproben zu widersprechen und ihre Vernichtung zu verlangen.

Ich habe eine Kopie der schriftlichen Studieninformation und der Einwilligungserklärung mit Versions-Datum 09.09.2021 erhalten.

Ich erkläre, dass ich freiwillig bereit bin, an der wissenschaftlichen Studie teilzunehmen.

Einverständnis zur Erhebung und Verarbeitung von Daten

Mir ist bekannt, dass bei der oben genannten Studie personenbezogene Daten, wie in der Studieninformation beschrieben, erhoben, pseudonymisiert (verschlüsselt) aufgezeichnet, auf elektronischen Datenträgern gespeichert und verarbeitet werden sollen. Die Verarbeitung der Daten erfolgt nach gesetzlichen Bestimmungen und setzt gemäß Art. 6 Abs. 1 lit. a, Art. 9 Abs. 2 lit. a der Datenschutz-Grundverordnung (DSGVO) folgende Einwilligungserklärung voraus.

Ich stimme der Erhebung und Verarbeitung meiner personenbezogenen Daten durch das Studienteam von Prof. Dr. med. Margitta Worm zum Zwecke der Durchführung der Studie freiwillig zu und darüber hinaus wie folgt:

Ich stimme insbesondere zu, dass die Studienergebnisse in *anonymer* Form, die keinen Rückschluss auf die meine Person zulässt, veröffentlicht werden. Mir ist bewusst, dass das Anonymisieren dazu führen kann, dass eine Rückverfolgung der Datenverarbeitung ausgeschlossen ist, so dass dann meine Rechte auf Auskunft, Berichtigung oder Löschung/Vernichtung nicht mehr durchgesetzt werden können.

Ich bin damit einverstanden, dass das für den Zweck der oben genannten Studie entnommene Blut, Hautabstriche, Hausstaub, Speichel- und Stuhlproben pseudonymisiert durch folgende Labor untersucht werden können:

- Allergologisches Forschungslabor der Kinderklinik; Charité - Universitätsmedizin Berlin; Augustenburger Platz 1, 13353 Berlin
- Klinik für Dermatologie, Venerologie und Allergologie; Charité - Universitätsmedizin Berlin; Charitéplatz 1, 10117 Berlin
- Institut für Mikrobiologie und Infektionsimmunologie; Hindenburgdamm 30, 12203 Berlin
- Max-Delbrück-Centrum; Charité - Universitätsmedizin Berlin, Robert-Rössle-Str. 10, 13092 Berlin
- Experimental and Clinical Research Center des Max-Delbrück-Centrum und der Charité; Lindenberger Weg 80, 13125 Berlin
- Berlin-Brandenburg Center für Regenerative Therapien (BCRT); Augustenburger Platz 1, 13353 Berlin
- Labor Berlin | Sylter Straße 2, 13353 Berlin
- Thermo Fisher Scientific, Servicelabor, Munzinger Straße 7, 79111 Freiburg

Weiterhin bin ich einverstanden, dass ein Teil meiner pseudonymisierten Bioproben für 10 Jahre nach Studienabschluss und Veröffentlichung der Ergebnisse für spätere (möglicherweise auch genetische) Analysen, in weiteren Projekten unter Wahrung des Datenschutzes gelagert werden dürfen:

☐ Ja ☐ Nein

Ich stimme zu, dass für den Zweck der oben genannten Studie die pseudonymisierten Studiendaten übermittelt werden an:

- Institut für Sozialmedizin, Epidemiologie und Gesundheitsökonomie; Charité - Universitätsmedizin Berlin; Charitéplatz 1, 10117 Berlin

- Institut für Physiologie, Charité - Universitätsmedizin Berlin; Charité Platz 1, 10117 Berlin
- Experimental and Clinical Research Center des Max-Delbrück-Centrum und der Charité; Lindenberger Weg 80, 13125 Berlin

Ich wurde darüber aufgeklärt, dass ich diese Einwilligung in die Datenverarbeitung jederzeit für die Zukunft widerrufen kann und dass der Widerruf die Rechtmäßigkeit der bereits erfolgte Datenverarbeitung nicht berührt.

Darüber hinaus habe ich zur Kenntnis genommen, dass ich mich zur Wahrnehmung meiner Rechte auf Auskunft in maschinenlesbarem Format, auf Berichtigung, Löschung/Vernichtung und Einschränkung der Verarbeitung an die Charité - Universitätsmedizin wenden kann, dort das Studienteam von Prof. Dr. med. M. Worm bzw. den Datenschutzbeauftragten und im Falle von Fragen und Einwendungen bezüglich der Verarbeitung meiner personenbezogenen Daten an die Beauftragte für Datenschutz und Informationssicherheit von Berlin, deren Kontaktdaten in der Studieninformation aufgeführt sind.

Im Rahmen eines Aufklärungsgesprächs hatte ich die Gelegenheit, hierzu Fragen zu stellen. Für die Entscheidung hatte ich ausreichend Zeit.

Eine Ausfertigung der Informationen und der Einwilligungserklärung habe ich erhalten.

Einwilligungserklärung zur Kontaktaufnahme bezüglich aufbauender Studien

Des Weiteren erkläre ich mich damit einverstanden, dass die Studienleitung mich gegebenenfalls bezüglich einer potentiellen Teilnahme an auf die aktuelle Studie aufbauende Folgestudien telefonisch oder per E-Mail kontaktieren darf.

☐ Ja ☐ Nein

Falls Ja. Ich möchte auf folgende Weise kontaktiert werden (es ist möglich alle Optionen auszufüllen):

☐ telefonisch unter: ______________________ ☐ per E-Mail unter: _____________________

☐ per Post unter: ____

Berlin, den ____

Unterschrift der Teilnehmerin/des Teilnehmers

Hiermit erkläre ich, die Teilnehmerin/den Teilnehmer am __________________ über Wesen, Bedeutung und Risiken der o.g. Studie mündlich und schriftlich aufgeklärt, alle Fragen zur Studie einschließlich der beabsichtigten Datenvereinbarung beantwortet und ihr/ihm eine Kopie der Studieninformation und der Einwilligungserklärung übergeben habe.

Berlin, den

………………………………………………………………………………….………

Unterschrift der/des aufklärenden Studienärztin/-arztes

Consent Form for parents

Einwilligungserklärung für Erziehungsberechtigte

für die Teilnahme an der Studie

Förderung der Toleranzentwicklung durch „Nicht-Vermeidung“ zur Verhinderung einer persistierenden Nahrungsmittelallergie

Hiermit erklären wir/ich

....	....	....
Vorname	Name	Geburtsdatum

....	....	....
Vorname	Name	Geburtsdatum

dass wir/ich durch Herrn/Frau ....
(Name des Studienarztes / der Studienärztin)

mündlich und schriftlich über das Wesen, die Bedeutung und die Risiken der wissenschaftlichen Untersuchungen im Rahmen der o.g. Studie informiert wurden und ausreichend Gelegenheit hatte, unsere Fragen mit dem Studienarzt/der Studienärztin zu klären.

Uns ist bekannt, dass wir das Recht haben, unsere Einwilligung jederzeit ohne Angabe von Gründen und ohne nachteilige Folgen zurückzuziehen und einer Weiterverarbeitung der Daten und Bioproben zu widersprechen und ihre Vernichtung zu verlangen.

Wir haben eine Kopie der schriftlichen Studieninformation und der Einwilligungserklärung mit Versions-Datum 09.09.2021 erhalten.

Wir erklären, dass wir freiwillig bereit sind, dass unser Kind

....	....	....
Vorname	Name	Geburtsdatum

an der wissenschaftlichen Studie teilnimmt.

Einverständnis zur Erhebung und Verarbeitung von Daten

Uns ist bekannt, dass bei der oben genannten Studie personenbezogene Daten, wie in der Studieninformation beschrieben, erhoben, pseudonymisiert (verschlüsselt) aufgezeichnet, auf elektronischen Datenträgern gespeichert und verarbeitet werden sollen. Die Verarbeitung der Daten erfolgt nach gesetzlichen Bestimmungen und setzt gemäß Art. 6 Abs. 1 lit. a, Art. 9 Abs. 2 lit. a der Datenschutz-Grundverordnung (DSGVO) folgende Einwilligungserklärung voraus.

Wir stimmen der Erhebung und Verarbeitung der personenbezogenen Daten unseres Kindes durch das Studienteam von Prof. Dr. med. Kirsten Beyer zum Zwecke der Durchführung der Studie freiwillig zu und darüber hinaus wie folgt:

Wir stimmen insbesondere zu, dass die Studienergebnisse in *anonymer* Form, die keinen Rückschluss auf die Person unseres Kindes zulässt, veröffentlicht werden. Uns ist bewusst, dass das Anonymisieren dazu führen kann, dass eine Rückverfolgung der Datenverarbeitung ausgeschlossen ist, so dass dann unsere Rechte auf Auskunft, Berichtigung oder Löschung/Vernichtung nicht mehr durchgesetzt werden können.

Wir sind damit einverstanden, dass das für den Zweck der oben genannten Studie entnommene Blut, Hautabstriche, Hausstaub, Speichel- und Stuhlproben pseudonymisiert durch folgende Labor untersucht werden können:

- Allergologisches Forschungslabor der Kinderklinik, Charité - Universitätsmedizin Berlin; Augustenburger Platz 1, 13353 Berlin
- Klinik für Dermatologie, Venerologie und Allergologie, Charité - Universitätsmedizin Berlin; Charitéplatz 1, 10117 Berlin
- Institut für Mikrobiologie und Infektionsimmunologie,Charité - Universitätsmedizin Berlin; Hindenburgdamm 30, 12203 Berlin
- Max-Delbrück-Centrum, Charité - Universitätsmedizin Berlin; Robert-Rössle-Str. 10, 13092 Berlin
- Experimental and Clinical Research Center des Max-Delbrück-Centrum und der Charité; Lindenberger Weg 80, 13125 Berlin
- Berlin-Brandenburg Center für Regenerative Therapien (BCRT); Augustenburger Platz 1, 13353 Berlin
- Labor Berlin | Sylter Straße 2, 13353 Berlin
- Thermo Fisher Scientific, Servicelabor, Munzinger Straße 7, 79111 Freiburg

Weiterhin sind wir einverstanden, dass ein Teil der pseudonymisierten Bioproben unseres Kindes für 10 Jahre nach Studienabschluss und Veröffentlichung der Ergebnisse für spätere (möglicherweise auch genetische) Analysen, in weiteren Projekten unter Wahrung des Datenschutzes gelagert werden dürfen:

☐ Ja ☐ Nein

Wir stimmen zu, dass für den Zweck der oben genannten Studie die pseudonymisierten Studiendaten übermittelt werden an:

- Institut für Sozialmedizin, Epidemiologie und Gesundheitsökonomie,
  Charité - Universitätsmedizin Berlin; Charitéplatz 1, 10117 Berlin
- Klinik für Dermatologie, Venerologie und Allergologie, Charité - Universitätsmedizin Berlin;
  Charitéplatz 1, 10117 Berlin
- Institut für Physiologie, Charité - Universitätsmedizin Berlin;
  Charitéplatz 1, 10117 Berlin
- Experimental and Clinical Research Center des Max-Delbrück-Centrum und der Charité;
  Lindenberger Weg 80, 13125 Berlin

Wir wurden darüber aufgeklärt, dass diese Einwilligung in die Datenverarbeitung jederzeit für die Zukunft widerrufen werden kann und dass der Widerruf die Rechtmäßigkeit der bereits erfolgte Datenverarbeitung nicht berührt.

Darüber hinaus haben wir zur Kenntnis genommen, dass wir uns zur Wahrnehmung des Rechtes auf Auskunft in maschinenlesbarem Format, auf Berichtigung, Löschung und Einschränkung der Verarbeitung an die Charité - Universitätsmedizin wenden können, dort den Studienleiter Prof. Dr. med. Kirsten Beyer bzw. den Datenschutzbeauftragten und im Falle von Fragen und Einwendungen bezüglich der Verarbeitung meiner personenbezogenen Daten an die Beauftragte für Datenschutz und Informationssicherheit von Berlin, deren Kontaktdaten in der Studieninformation aufgeführt sind.

Im Rahmen eines Aufklärungsgesprächs hatten wir die Gelegenheit, hierzu Fragen zu stellen. Für die Entscheidung hatten wir ausreichend Zeit.

Eine Ausfertigung der Informationen und der Einwilligungserklärung haben wir erhalten.

Einwilligungserklärung zur Kontaktaufnahme bezüglich aufbauender Studien

Des Weiteren erklären wir uns damit einverstanden, dass die Studienleitung uns gegebenenfalls bezüglich einer potentiellen Teilnahme an auf die aktuelle Studie aufbauende Folgestudien telefonisch, per E-Mail oder postalisch kontaktieren darf.

☐ Ja ☐ Nein

Falls Ja. Wir möchten auf folgende Weise kontaktiert werden (es ist möglich alle Optionen auszufüllen): ☐ telefonisch unter: ______________________ ☐ per E-Mail unter: ______________________

☐ per Post unter: ____

Sorgerecht:

☐ Gemeinsame Ausübung des Sorgerechts

☐ Alleinige Ausübung des Sorgerechts. Hiermit bestätige ich die alleinige Ausübung des Sorgerechts für mein oben genanntes Kind.

Berlin, den ____
Unterschrift der Mutter

Berlin, den ____
Unterschrift des Vaters

Hiermit erkläre ich, die Eltern des o.g. Kindes am ___________________ über Wesen, Bedeutung und Risiken der o.g. Studie mündlich und schriftlich aufgeklärt, alle Fragen zur Studie einschließlich der beabsichtigten Datenvereinbarung beantwortet und ihnen eine Kopie der Studieninformation und der Einwilligungserklärung übergeben habe.

Berlin, den ……………………………………………………………………………………………………..
Name Unterschrift der/des aufklärender/n Studienärztin/-arztes
